# Metabolic Reprogramming of a Phenolic Acid by a Plant P450 Monooxygenase Reverses Bacterial Immunosuppression

**DOI:** 10.1111/pbi.70660

**Published:** 2026-03-31

**Authors:** Hua Wang, Xiaoshuang Peng, Xinrui Wang, Ruiyu Yang, Tao Liu, Huidi Liu, Chao Zhao, Hong Zhou, Liang Yang, Shili Li, Wei Ding

**Affiliations:** ^1^ Laboratory of Natural Products Pesticides, College of Plant Protection Southwest University Chongqing China; ^2^ Biotechnology Laboratory, Shijiazhuang Institute of Pomology Hebei Academy of Agriculture and Forestry Sciences Shijiazhuang China

**Keywords:** NLR genes, P450 monooxygenase, plant immunity suppression, *Ralstonia solanacearum*, TMV, veratric acid

## Abstract

Soil acidification often exacerbates plant diseases caused by soil‐borne pathogens like *Ralstonia solanacearum* , but the underlying molecular mechanisms remain elusive. This study unveils a sophisticated metabolic game in the tobacco‐ *R. solanacearum* pathosystem, where the pathogen manipulates host metabolism to suppress immunity, and the plant counteracts by enzymatically reprogramming a key metabolic signal. Using multi‐omics approaches, we discovered that *R. solanacearum* infection induces a significant accumulation of veratric acid (VA) in tobacco. We demonstrated that VA acts as a potent immunosuppressant rather than a nutrient for the pathogen. It broadly inhibits plant pattern‐triggered immunity, including flg22‐induced ROS burst, and transcriptionally represses a suite of nucleotide‐binding leucine‐rich repeat (NLR) receptors, crucially including *NtG28897* (*NtTAO1*). Silencing of *NtTAO1* confirmed its pivotal role in resistance against Tobacco Mosaic Virus (TMV). Facing this metabolic sabotage, tobacco engages a counter‐defence mechanism. We identified a specific cytochrome P450 monooxygenase (CYP86A22) that catalyses the conversion of the disease‐promoting VA into vanillic acid (VanA). Transient overexpression of this P450 in *Nicotiana benthamiana* enhanced the in vivo conversion of VA to VanA. Crucially, this enzymatic conversion conferred strong resistance against TMV, whereas the P450 or VA alone did not. Our findings reveal a novel plant immune strategy ‘metabolic signal reprogramming’ where a P450 enzyme detoxifies a susceptibility metabolite into a defensive compound. This work provides a new paradigm for plant–pathogen interactions and identifies promising targets for metabolic engineering or green chemical strategies to achieve sustainable disease control.

## Graphical abstract

*Ralstonia solanacearum* suppresses P450 activity to block VA‐to‐VanA conversion, leading to VA accumulation and compromised immunity.

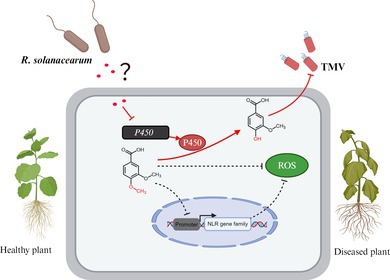

## 1 Introduction

The health and productivity of plants are constantly threatened by soil‐borne pathogens, with their susceptibility being significantly shaped by the rhizosphere environment, particularly soil pH (Wang and Kuzyakov 2024). A prime example is *Ralstonia solanacearum* , a devastating phytopathogen that causes bacterial wilt in over 300 plant species worldwide (Jiang et al. 2017; Wang et al. 2023). A key facet of its ecological success lies in its sophisticated pathogenic strategies, including chemotaxis towards root‐derived organic acids, which facilitates host colonisation (Vailleau and Genin 2023). Notably, acidic soils are frequently observed to exacerbate the infectivity of *R. solanacearum* and other soil‐borne pathogens, as evidenced in crops such as tobacco (Prusky and Yakoby 2003). This strong correlation suggests a critical, yet poorly understood, interplay between environmental acidification and microbial virulence, particularly regarding the molecular mechanisms by which acidic conditions enhance plant susceptibility (Liu et al. 2022; Jia et al. 2024; Singh et al. 2025). However, the molecular mechanisms through which acidic conditions enhance plant susceptibility remain largely elusive.

Phenolic acids are key mediators at the plant–microbe interface, with their profiles and activities often regulated by soil pH. Their role in plant–pathogen interactions is context‐dependent, exhibiting a classic ‘double‐edged sword’ character: while some, such as salicylic acid, ferulic acid and caffeic acid, contribute to chemical defence or systemic immunity (Li et al. 2021; Spoel and Dong 2024; Ma et al. 2025). Others can be exploited by pathogens as carbon sources or virulence signals (Feng et al. 2025; Li et al. 2023; Yang et al. 2024; Zhao et al. 2024). This functional duality highlights that the outcome of phenolic acid accumulation depends on the specific metabolite, pathogen and physiological context. Therefore, identifying which phenolic acids accumulate during *R. solanacearum* infection and defining their functional role represents a critical first step.

Crucially, whether a phenolic acid acts as a defence signal or a virulence cue depends ultimately on how it is perceived and processed within the plant immune system. This immune system, comprising pattern‐triggered (PTI) and effector‐triggered immunity (ETI), is a frequent target of pathogen suppression. For example, *Pseudomonas syringae* employs the effector HopAI1 to directly inactivate Arabidopsis MAP kinases MPK3 and MPK6, effectively blocking PAMP/MAMP‐triggered immune responses (Zhang et al. 2007). Another effector from *P. syringae* , HopM1, disrupts salicylic acid (SA)‐dependent defence mechanisms (DebRoy et al. 2004). These findings unequivocally demonstrate that phytopathogens utilise effector proteins to subvert host disease resistance. Crucially, the efficacy of this immune system is not solely determined by protein–protein interactions but is also profoundly shaped by the dynamic metabolic landscape within the plant (Tsai and Schmidt 2021). A compelling, yet underappreciated, dimension of this interplay is the enzymatic conversion of phenolic and other defensive compounds, which can fundamentally alter the outcome of plant–pathogen interactions. For instance, the level of acetosyringone, a phenolic compound, is elevated during PTI in tobacco, where it acts synergistically with plant‐derived hydrogen peroxide and peroxidase to exert a broad‐spectrum antibacterial effect, primarily by depolarising bacterial membranes (Szatmari et al. 2021). These findings highlight the central role of specific compounds, particularly phenolic acids, in shaping plant immune outcomes. A key uncharted frontier is whether enzymes can directly ‘reprogramme’ the immune signal by converting a disease‐promoting phenolic acid into a beneficial, directly antimicrobial one.

Despite these advances, critical knowledge gaps persist. The origin of pathogen‐favourable acidification—whether host‐derived or actively orchestrated by the pathogen—remains ambiguous. Moreover, the molecular mechanisms linking specific pathogen‐induced metabolites to the suppression of host immune components are still elusive. Most importantly, it is unknown whether plants possess counter‐strategies to reprogramme such disease‐promoting metabolic signals, and if such strategies involve metabolic conversion by enzymes like P450 monooxygenases.

Cytochrome P450 monooxygenases constitute one of the largest enzyme families in plants and are instrumental in synthesising a vast array of specialised metabolites, including many with known roles in defence, such as phytoalexins, lignin precursors and defence hormones (Hansen et al. 2021). Owing to their remarkable substrate versatility and regio‐selectivity, P450 enzymes serve as ideal catalysts for the precise modification of bioactive molecules (Mei et al. 2024). While the role of P450s in detoxifying xenobiotic compounds is well established, a far less explored frontier is their potential to ‘detoxify’ or ‘reprogramme’ host‐derived metabolites—converting endogenous molecules with disease‐promoting potential into beneficial compounds (Dimaano and Iwakami 2021). A pivotal question thus arises: Can P450‐mediated hydroxylation or demethylation transform a disease‐promoting phenolic acid into a defensive one? Such a mechanism would represent a sophisticated plant countermeasure, moving beyond the mere production of antimicrobials to the active remodelling of the chemical landscape shaped by the pathogen.

In this study, we combine metabolomic, transcriptomic and molecular plant pathological approaches to investigate the metabolic interplay in the tobacco– *R. solanacearum* pathosystem. We hypothesise that pathogen infection reprogrammes host metabolism to accumulate specific phenolic acids (e.g., veratric acid), which in turn suppress plant immunity by interfering with hormonal signalling networks. Furthermore, we postulate that the plant engages a counter‐defence mechanism by enzymatically converting this disease‐promoting metabolite into a defensive compound (e.g., vanillic acid), via the action of a specific P450 monooxygenase. This study aims to: (1) identify key immunosuppressive phenolic acids accumulated during *R. solanacearum* infection; (2) elucidate the molecular mechanisms by which they disrupt plant immune signalling; and (3) reveal the biochemical pathway of the plant's metabolic countermeasure mediated by a P450 enzyme. Our findings aim to elucidate a novel mechanism of pathogen‐induced immune suppression via metabolic manipulation and to reveal a corresponding plant enzymatic countermeasure, providing new theoretical insights into the environmental modulation of plant immunity and scientific basis for developing sustainable disease management strategies in acid‐sensitive agroecosystems.

## 2 Results

### 2.1 *R. solanacearum* Drives Contrasting pH Shifts in *Nicotiana tabacum* Systems

**FIGURE 1:**
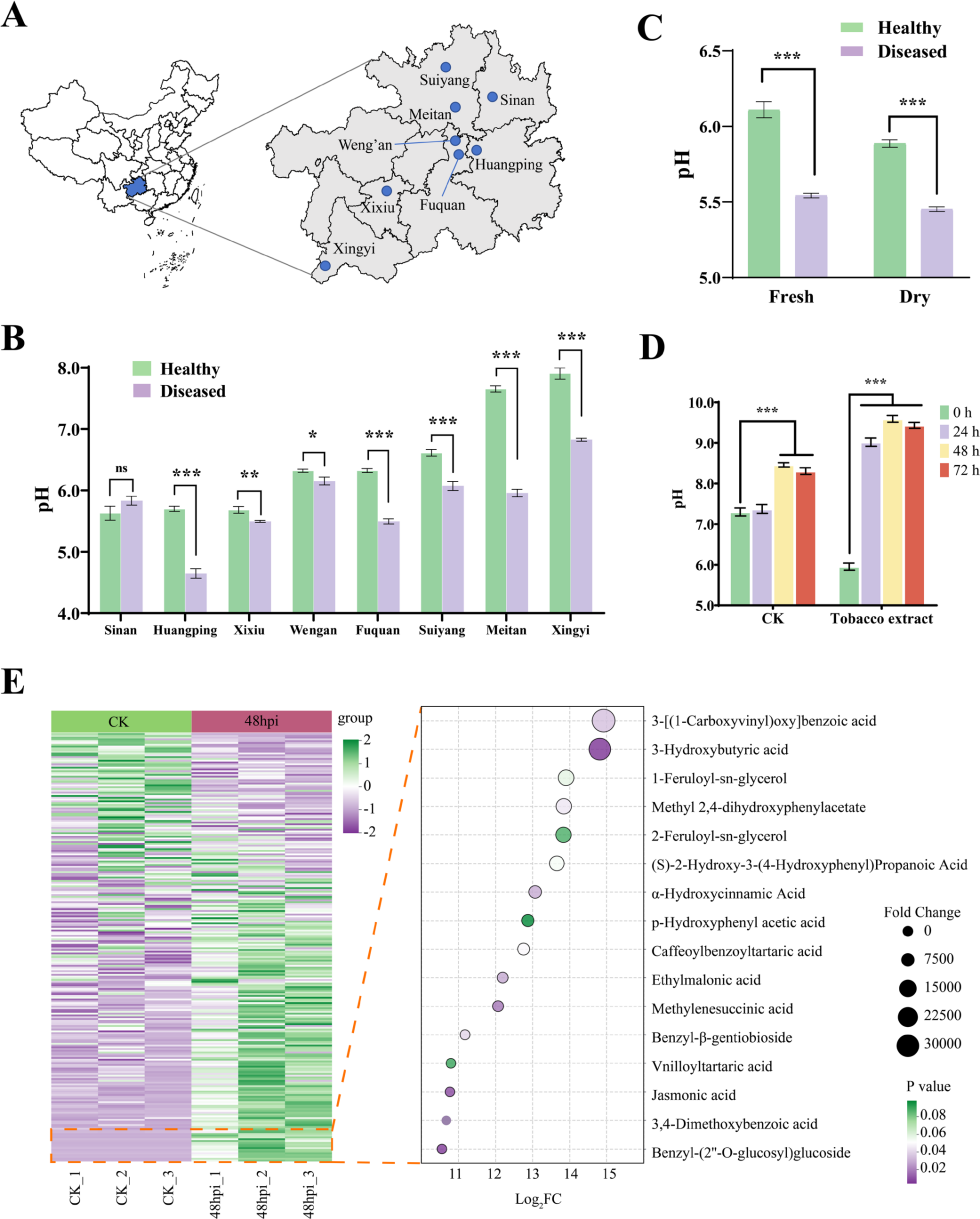
pH differences between diseased and healthy tobacco systems. (A) Sampling locations in Guizhou Province, China. (B) pH comparison between adjacent diseased and healthy field plots in Guizhou. (C) pH differences between symptomatic and asymptomatic tissues in controlled inoculation experiments. (D) pH trajectories of *Ralstonia solanacearum* cultures in *Nicotiana tabacum* intercellular fluid versus control medium. (E) Metabolomic differences between diseased and non‐diseased tissue (*p < 0.05; **p < 0.01; ***p < 0.001; error bars = SD).

Soil pH analysis in adjacent diseased and healthy *N. tabacum* ‐growing plots across Guizhou Province revealed significantly lower pH values in diseased areas compared to healthy counterparts (*p* < 0.05). This consistent pattern was disrupted in Sinan County, Tongren City, where no statistically significant pH difference was observed between diseased and healthy plots (Figure 1A,B). Controlled inoculation experiments with *R. solanacearum* demonstrated that infected *N. tabacum* plants exhibited significantly lower pH levels compared to asymptomatic plants (*p* < 0.001) (Figure 1C). Subsequent investigation using sterilised plant tissue (intercellular fluid) inoculated with the pathogen revealed contrasting pH dynamics: the bacterial culture medium displayed increased alkalinity relative to baseline measurements (Figure 1D).

To characterise the key compounds responsible for pH reduction, this study utilised liquid chromatography‐mass spectrometry (LC/MS) for qualitative and quantitative analysis of relevant compounds in samples. Results at 48 h post‐inoculation showed a total of 243 organic acid compounds identified, of which 161 were phenolic acids (shown in Supporting Information and Figure S1 for 96 h). Differential expression analysis further revealed that 66 organic acids were significantly upregulated (*p* < 0.05), while only three exhibited significant downregulation (Figure 1E). Notably, 16 acids including VA (3,4‐dimethoxybenzoic acid) showed particularly pronounced upregulation (Log_2_FC > 10). These findings indicate that the significant accumulation of these key compounds in diseased *N. tabacum* tissues serves as a core driving factor for the decrease in microenvironmental pH.

### 2.2 Contrasting Effects of Organic Acids on *R. solanacearum* ‐Induced Disease Progression

**FIGURE 2:**
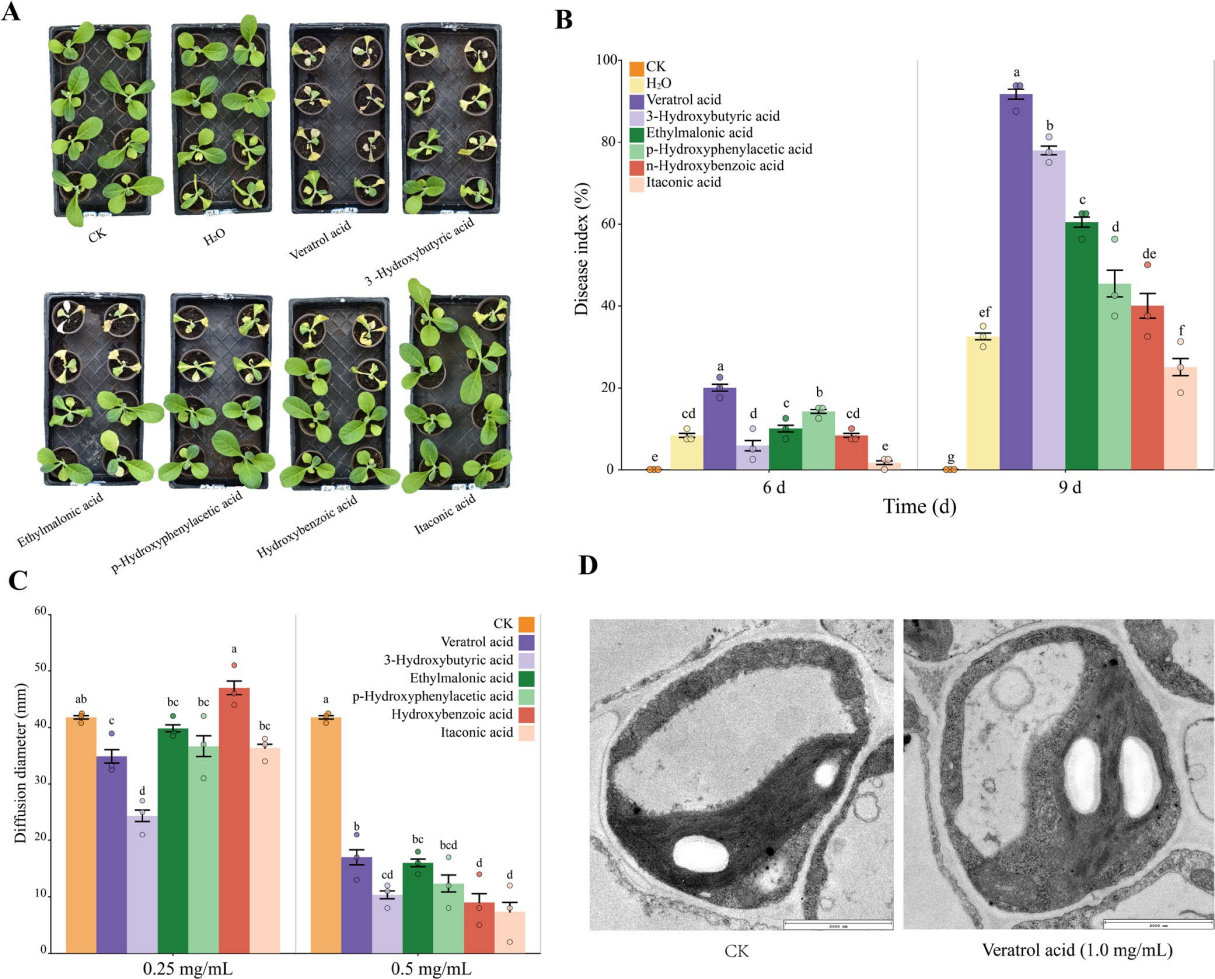
Contrasting effects of organic acids on *Ralstonia solanacearum* ‐induced disease progression. (A, B) Promotion of bacterial wilt disease by different phenolic acids (at 1 mg/mL); (C) Dose‐dependent inhibition of bacterial swimming motility, representative swimming zones of *R. solanacearum* under 0.25 mg/mL, 0.5 mg/mL and control treatments (CK). (D) Ultrastructural preservation of *Nicotiana tabacum* leaf cells. (1 mg/mL VA spraying) (CK, control group, different letters represent significant differences among treatments at the same time point, error bars = SD, *p* < 0.05).

To investigate the contribution of organic acids to *R. solanacearum* infection, six upregulated organic acids in diseased *N. tabacum* tissues were selected for disease promotion assays. By 9 days post‐inoculation (dpi), the disease index of the water control reached 32.5%. Notably, VA (93.75%), 3‐hydroxybutyric acid (81.25%) and ethylmalonic acid (56.25%) significantly accelerated disease progression, with disease indices exceeding the control by 61.25%, 48.75% and 23.75%, respectively. In contrast, itaconic acid suppressed disease development, yielding a markedly lower disease index (18.75%) (Figure 2A,B). Early‐stage observations at Day 6 revealed that VA already induced a significantly higher disease index (20%) compared to the water control (7.5%). These findings demonstrate that VA, but not all tested organic acids (such as itaconic acid can suppress disease), enhances *R. solanacearum* ‐induced wilt progression, whereas itaconic acid exhibits inhibitory effects on disease onset.

### 2.3 Effects of Tested Compounds on Pathogen and *N. tabacum*

At a concentration of 0.5 mg/mL, the tested compounds significantly inhibited the swimming motility of *R. solanacearum* (*p* < 0.05). This inhibitory effect was markedly stronger than that observed at 0.25 mg/mL, demonstrating clear dose dependency (Figure 2C). Data from the 1 mg/mL treatment were excluded from motility analysis due to complete suppression of bacterial growth (Table S1). Transmission electron microscopy revealed that *N. tabacum* leaf cells treated with 1 mg/mL VA maintained intact ultrastructural features of mitochondria and chloroplasts, with no signs of vacuolation or membrane damage compared to controls (Figure 2D). Notably, although these findings would suggest that VA treatment should confer resistance against bacterial wilt in *N. tabacum* , no such protective effects were observed in our previous inoculation assays—revealing an apparent paradox between the compound's in vitro antimicrobial activity and its in planta efficacy.

Integrated analysis indicates that these compounds effectively inhibit the growth of *R. solanacearum* under in vitro conditions but do not significantly suppress normal plant growth and development, suggesting that they may not be metabolites produced by *R. solanacearum* itself.

### 2.4 VA Enhances Pathogenicity of TMV and *P. syringae* pv. *tabaci*

**FIGURE 3:**
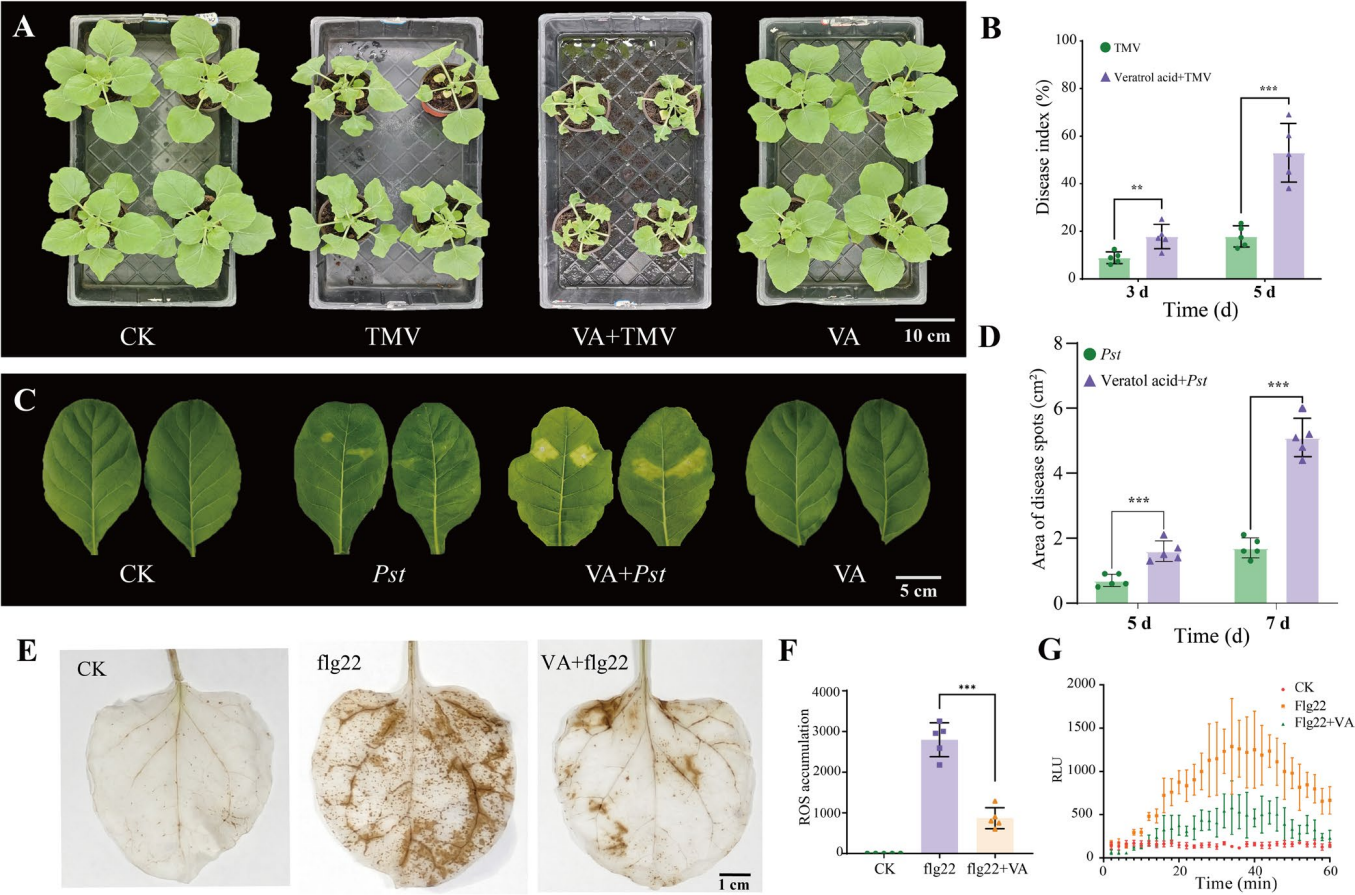
Veratric acid enhances tobacco viral‐bacterial diseases. (A, B) Symptoms of *Nicotiana benthamiana* leaves inoculated with tobacco mosaic virus (TMV) at 5 dpi, showing enhanced disease severity in the VA treatment group compared to the control group. Lesion phenotypes on *N. benthamiana* leaves infected with *Pseudomonas syringae* pv. *tabaci* (*Pst*) at 7 dpi, with larger lesion areas observed in the VA‐treated plants than in the controls. (C, D) Quantitative analysis of disease indices (for TMV infection at 3 and 5 dpi) and lesion areas (for *Pst* infection at 5 and 7 dpi) in VA‐treated and control plants. (E) DAB staining images depicting reactive oxygen species (ROS) accumulation in *N. benthamiana* leaves under three treatments: CK (untreated control), flg22 (flg22 treatment alone) and VA + flg22 (pretreated with VA followed by flg22 spray). (F) Relative staining intensity of DAB shown in (E), quantified using ImageJ software. (G) Quantitative analysis of flg22‐induced ROS burst measured by luminol‐based chemiluminescence assay under the same three treatments. Data are presented as mean ± standard deviation (*n* = 5). Asterisks indicate significant differences between groups (**p < 0.05; ***p < 0.001; Student's *t*‐test).

Based on the disease‐promoting effect of VA on bacterial wilt, this study further investigated its impacts on tobacco diseases caused by tobacco mosaic virus (TMV) and the *N. tabacum* wildfire bacterium ( *P. syringae* pv. *tabaci*). For TMV infection, the disease index of *Nicotiana benthamiana* treated with VA was 17.8 at 3 days post‐inoculation (dpi), compared with 8.9 in the control group. By 5 dpi, the disease index in the treatment group increased to 53.0, which was significantly higher than the control value of 17.8, indicating an expanding gap over time (Figure 3A,B). For infection by the tobacco wildfire bacterium, the lesion area in the treatment group was 1.7 cm^2^ at 5 dpi, compared with 0.7 cm^2^ in the control group; this area further increased to 5.1 cm^2^ at 7 dpi, significantly larger than the control value of 1.6 cm^2^, demonstrating that lesion expansion in the treatment group was significantly faster than in the control group (Figure 3C,D).

These observations at different times indicate that the disease‐promoting effect of VA on pathogens exhibits a time‐dependent cumulative pattern. This effect is not limited to the single *R. solanacearum* strain causing bacterial wilt, but also significantly promotes the early infection process of TMV (with enhanced pathogenicity already evident at 3 dpi) and the lesion expansion caused by *P. syringae* pv. *tabaci*. Based on these findings, we speculate that VA may reduce plant resistance to multiple pathogens at the early stage of disease development by affecting the plant disease resistance mechanism, thereby exacerbating symptom progression.

Further experiments verified the impact of VA on reactive oxygen species (ROS) accumulation in *N. benthamiana* leaves. Results showed that ROS accumulation was obstructed in *N. benthamiana* leaves treated with VA (Figure 3E–G). This indicates that VA can inhibit the ROS accumulation process induced by flg22, consequently suppressing plant resistance to diseases.

### 2.5 Transcriptional Repression of NLR Genes by VA Impairs Plant Defence Responses

**FIGURE 4:**
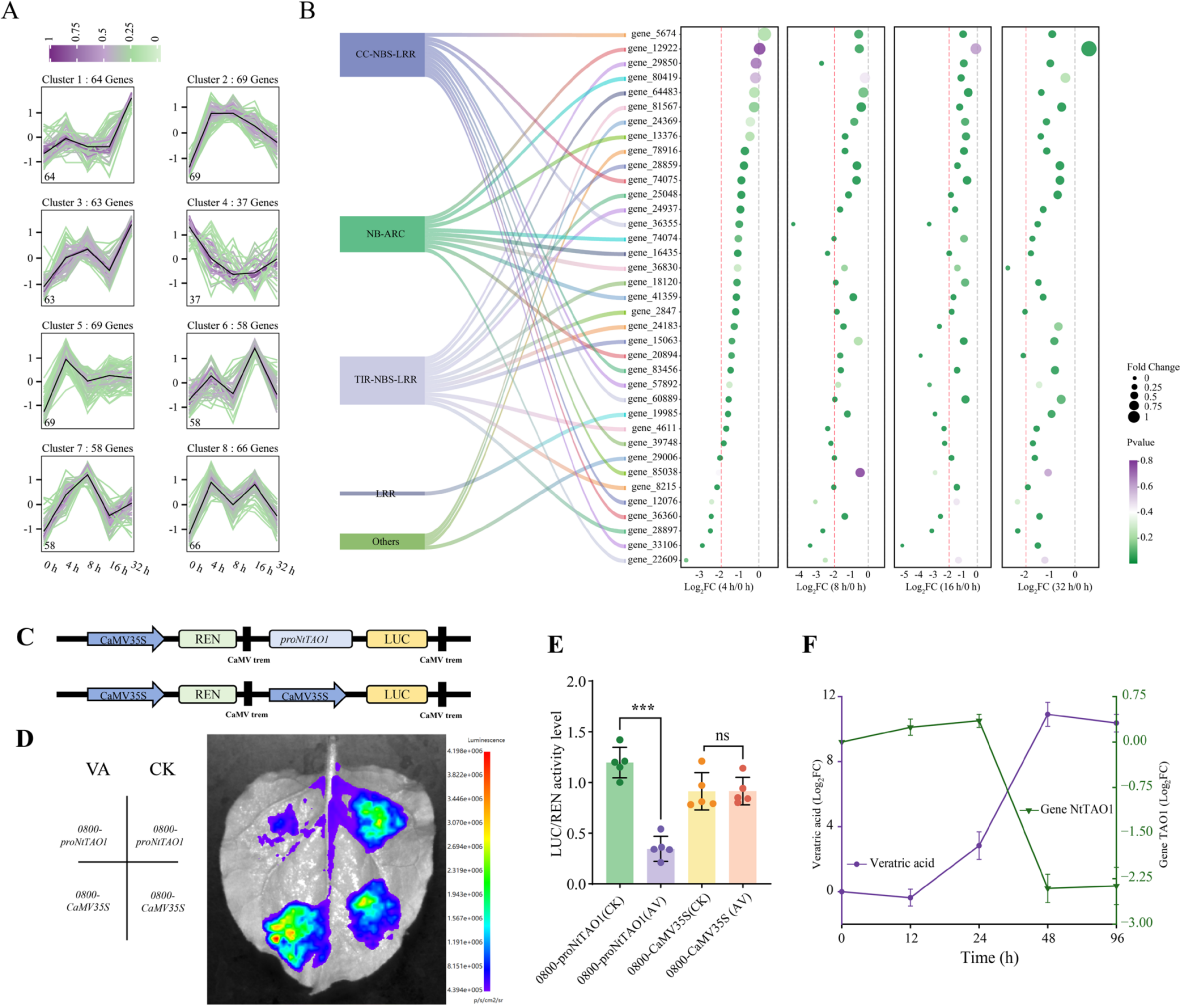
Time‐course gene expression and pathway enrichment after veratric acid treatment. (A) Time‐course gene enrichment trend analysis after VA treatment. Membership values (colour gradient) indicate the probability or weight of a DEG (differentially expressed gene) belonging to a specific cluster in fuzzy clustering. Samples are grouped by treatment conditions, and data are normalised by *Z*‐score. (B) Pathway enrichment analysis of gene Cluster 4 based on Domain. Results of gene enrichment difference analysis are shown in the right bubble plot, where the colour gradient reflects the significance of intergroup differences (*p* value), and bubble size represents the fold change of genes between groups. (C–E) Dual‐luciferase reporter assays revealed that the activity of the *proNtTAO1* (*NtG28897*) promoter, which drives the expression of *NtTAO1* in *Nicotiana tabacum* , was significantly repressed by VA treatment (***p < 0.001; Student's *t*‐test; ns: *p* > 0.05). (F) Changes in the relative contents of VA and *NtTAO1* in *N. tabacum* post *Ralstonia solanacearum* inoculation. Time‐series data of VA content and *NtTAO1* expression were measured. Data are shown as mean ± SD, grouped by time. A line graph depicts the trends, with the *y*‐axis for relative content/expression and the *x*‐axis for time after inoculation.

Transcriptomic analysis following VA treatment identified 484 differentially expressed genes (DEGs) associated with plant disease resistance. Fuzzy clustering of these DEGs revealed 37 genes with continuous downregulation over time, grouped into Cluster 4 (Figure 4A). Functional annotation showed that all Cluster 4 genes belonged to the nucleotide‐binding leucine‐rich repeat (NLR) gene family, a critical class of plant immune receptors essential for pathogen recognition and activation of defence responses.

Pathway enrichment analysis of Cluster 4 (Figure 4B) highlighted significant downregulation of NLR‐mediated defence pathways. Among these, *NtTAO1* (*NtG28897*; Figure S5) exhibited the most pronounced and consistent transcriptional repression, with fold changes below log_2_FC < −2 across all four time points analysed, indicating a robust and sustained reduction in expression. VA treatment induces transcriptional repression of NLR family genes, particularly *NtTAO1*, potentially compromising plant disease resistance mechanisms. Dual‐luciferase reporter assays revealed that the activity of the *proNtTAO1* promoter, which drives the expression of *NtTAO1* in *N. benthamiana*, was significantly repressed by VA treatment. Specifically, the LUC/REN ratio in VA‐treated plants was 0.34, significantly lower than that in untreated plants (1.21, *p* < 0.001). In the control groups (solvent‐treated and untreated), the LUC/REN ratios were 0.93 and 0.96, respectively, showing no statistical difference (*p* > 0.05). These results indicate that VA specifically suppresses the transcriptional activation of *proNtTAO1* (Figure 4C–E). A subset of these genes was selected for qRT‐PCR validation (Figures S2 and S3); the GO analysis and volcano plots at each time point are provided at Figure S4.

To investigate the temporal order of VA accumulation and *NtTAO1* downregulation following *R. solanacearum* infection, relative expression levels of both were measured in identical samples at sequential time points post‐inoculation (hpi). Quantitative analysis revealed that VA levels began to increase significantly at 12 hpi, whereas the expression of *NtTAO1* exhibited a notable downward trend starting at 24 hpi. This temporal separation of responses—with VA elevation preceding *NtTAO1* suppression—suggests a causal relationship in the natural infection process.

### 2.6 VA Differentially Affects Salicylic Acid and Jasmonic Acid Accumulation

To further explore the mechanism underlying VA‐mediated immune suppression, we quantified the endogenous levels of salicylic acid (SA) and jasmonic acid (JA) in *N. benthamiana* following VA treatment. As shown in Figure S16, exogenous application of VA significantly reduced SA accumulation compared to the mock control, whereas JA levels remained largely unchanged. These results suggest that VA may suppress plant immunity at least in part through downregulation of the SA pathway, while not affecting the JA pathway. The differential impact on these defence hormones is consistent with the observed suppression of NLR gene expression and enhanced disease susceptibility.

### 2.7 Silencing of *NtTAO1* Compromises *N. benthamiana* Resistance to *R. solanacearum* and TMV

**FIGURE 5:**
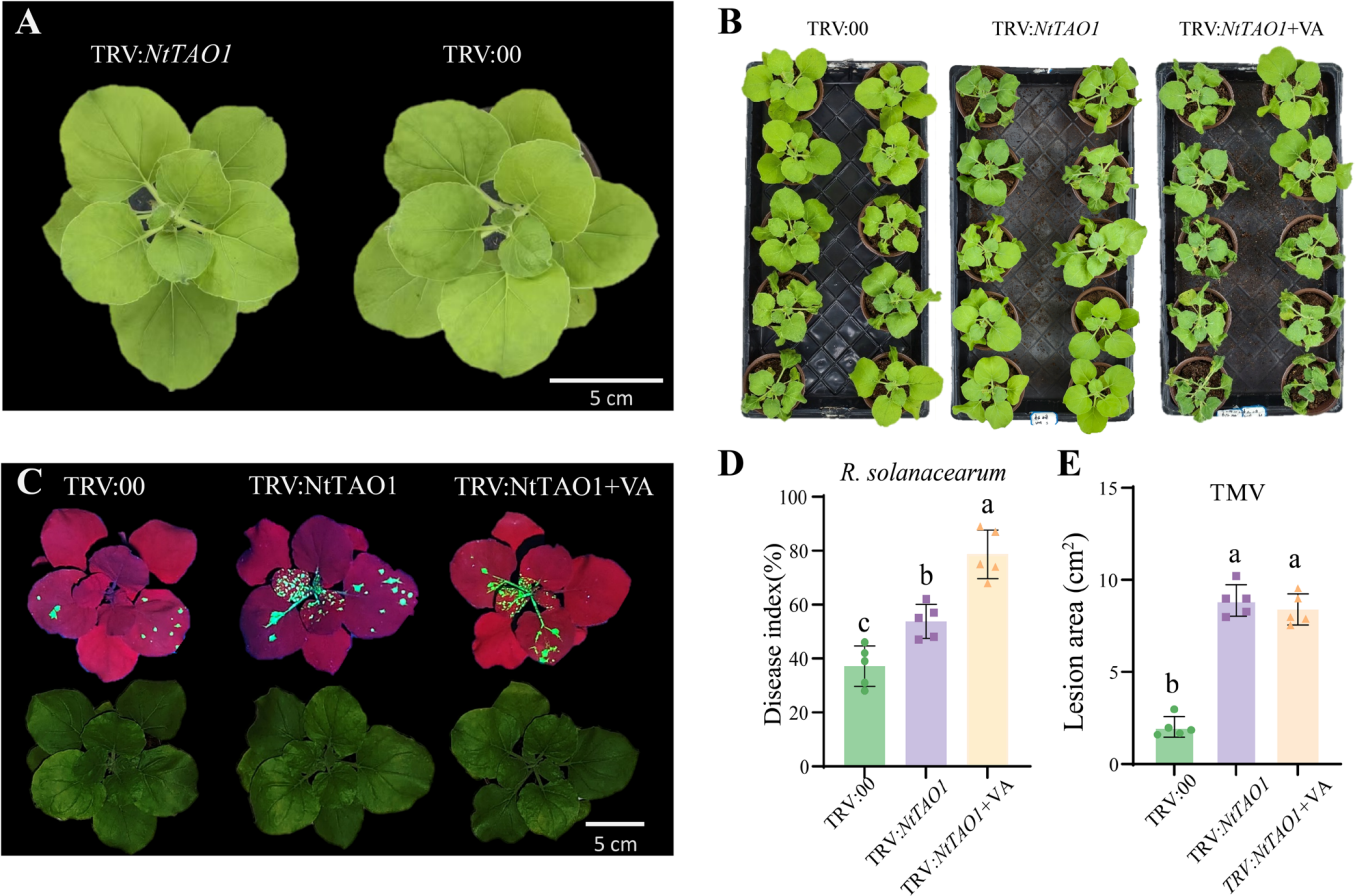
Silencing of *NtTAO1* enhances *Nicotiana benthamiana* susceptibility to TMV. (A) *N. benthamiana* with *NtTAO1* gene‐silenced (TRV: *NtTAO1*) and control plants (TRV:00). (B, D) Inoculation of *N. benthamiana* with *Ralstonia solanacearum* (bacterial wilt pathogen) under different treatments: TRV: *NtTAO1* (gene‐silenced) and TRV: *NtTAO1* + VA (veratric acid‐pretreated). (C, E) Inoculation of *N. benthamiana* with fluorescent TMV (visualising viral infection) under different treatments: TRV:00 (control), TRV: *NtTAO1* (gene‐silenced) and TRV: *NtTAO1* + VA (veratric acid treatment). Lowercase letters indicate significant differences between groups (*p* < 0.001).

In *R. solanacearum* infection assays, silencing of *NtTAO1* significantly enhanced plant susceptibility, with the disease index increasing to 53.8% compared to 37.2% in the control plants (Figure 5B,D). Critically, the application of VA to *NtTAO1*‐silenced plants drove the disease index even higher, to 78.6%. This additive effect demonstrates that VA must be targeting additional components of the plant immune system beyond *NtTAO1* to achieve full immunosuppression. In contrast, a distinct mechanism was observed for TMV. Following virus‐induced gene silencing (VIGS) of *NtTAO1*, pathogenicity analysis revealed significantly enhanced susceptibility, with the viral infection area reaching 8.88 cm^2^, 4.27‐fold larger than that in control TRV:00 plants (2.08 cm^2^, Figure 5C,E). However, spraying VA on *NtTAO1*‐silenced plants had no significant effect on TMV infection progression (infection area: 8.39 cm^2^). This indicates that *NtTAO1* is the primary and likely primary target for VA‐mediated suppression of TMV resistance. In conclusion, while *NtTAO1* is part of a broader network targeted by VA to promote bacterial wilt, it serves as the key factor for VA‐induced susceptibility to TMV.

### 2.8 Specific P450 Enzyme CYP86A22 Catalyses the Conversion of VA to VanA

**FIGURE 6:**
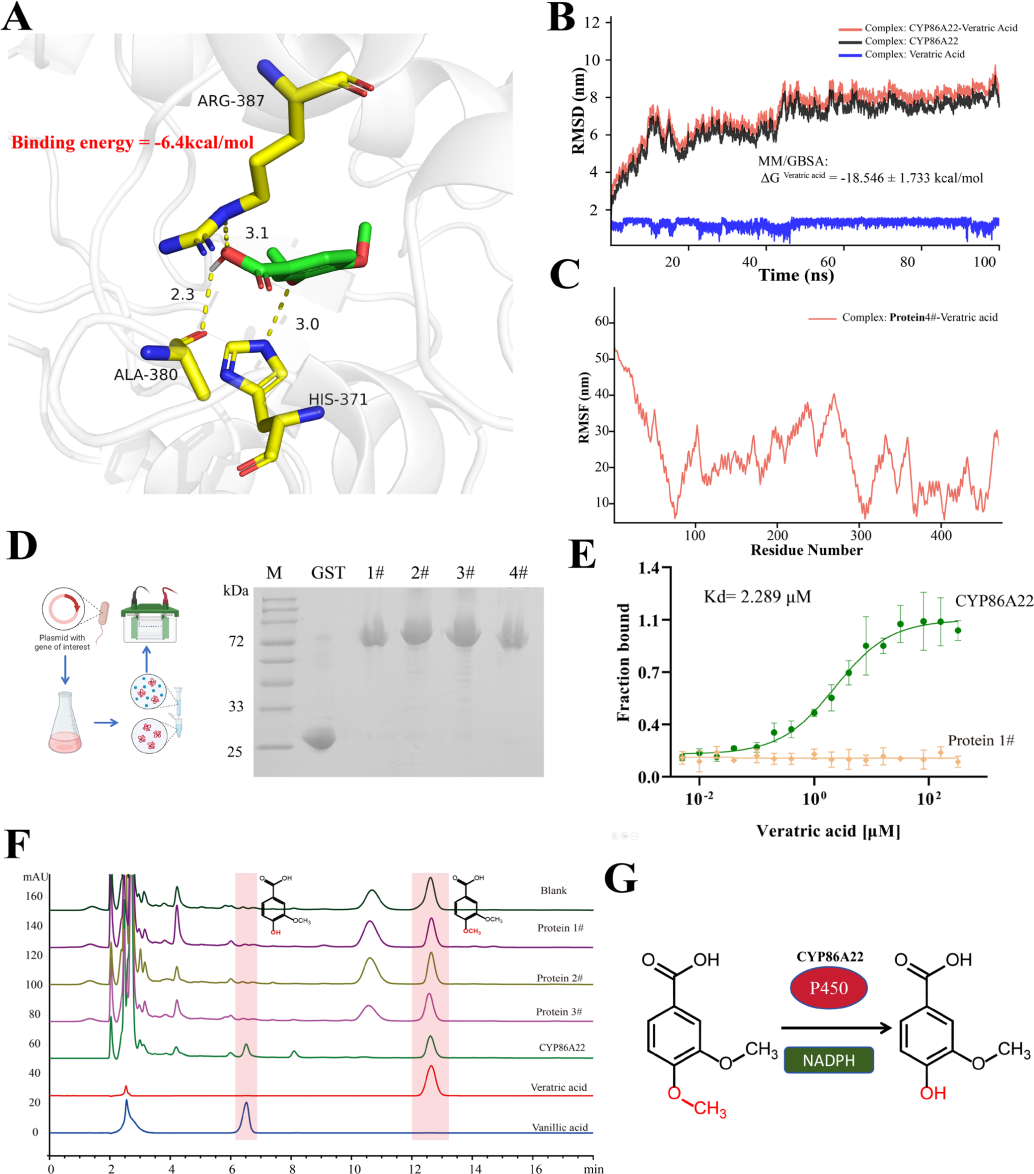
Specific cytochrome P450 enzyme catalyses the directed conversion of veratric acid to vanillic acid. (A) Molecular docking results demonstrate that CYP86A22 protein possesses specific binding capability with veratric acid, with a binding free energy of −6.4 kcal/mol. (B, C) Molecular dynamics simulation analysis of the CYP86A22‐veratric acid complex. The binding free energy (Δ*G* binding) was −18.546 ± 1.733 kcal/mol; root mean square fluctuation (RMSF) analysis indicates that the protein–ligand complex maintained high stability during the simulation. (D) Purification results of the four candidate P450 recombinant proteins, showing clear bands at the expected molecular weights. (E) Binding affinity between CYP86A22 and veratric acid measured by microscale thermophoresis (MST). The dissociation constant (*K*_d_) of protein 4# with veratric acid was 2.289 μM, indicating strong binding capacity. In contrast, the control protein 1# showed no detectable binding. (F) High‐performance liquid chromatography (HPLC) analysis of the catalytic activities of different P450 enzymes. Only CYP86A22 catalysed the production of VanA from veratric acid; no VanA was detected in reaction systems containing the other three enzymes. (G) In vitro catalytic system validation confirms that the specific P450 enzyme effectively converts veratric acid—accumulated in *Nicotiana benthamiana* during *Ralstonia solanacearum* infection—to VanA in the presence of NADPH.

Based on the transcriptomic analysis of *N. benthamiana* sprayed with VA, we identified a set of upregulated genes (Figure S6). The corresponding proteins were modelled using AlphaFold2, and their binding capabilities with VA were assessed, as summarised in Table S3. Four candidate proteins (1#–4#) with high docking scores were selected for further experimental validation (Figure 6, Figures S7–S10). Among these, the CYP86A22 (P450 4#)–VA complex was subjected to molecular dynamics simulation analysis. Further characterisation of the CYP86A22–VA complex by molecular dynamics simulation confirmed its strong and stable interaction, with a calculated binding free energy (Δ*G* binding) of −18.546 ± 1.733 kcal/mol. Root mean square fluctuation (RMSF) analysis indicated that the protein–ligand complex maintained high structural stability throughout the simulation trajectory.

**FIGURE 7:**
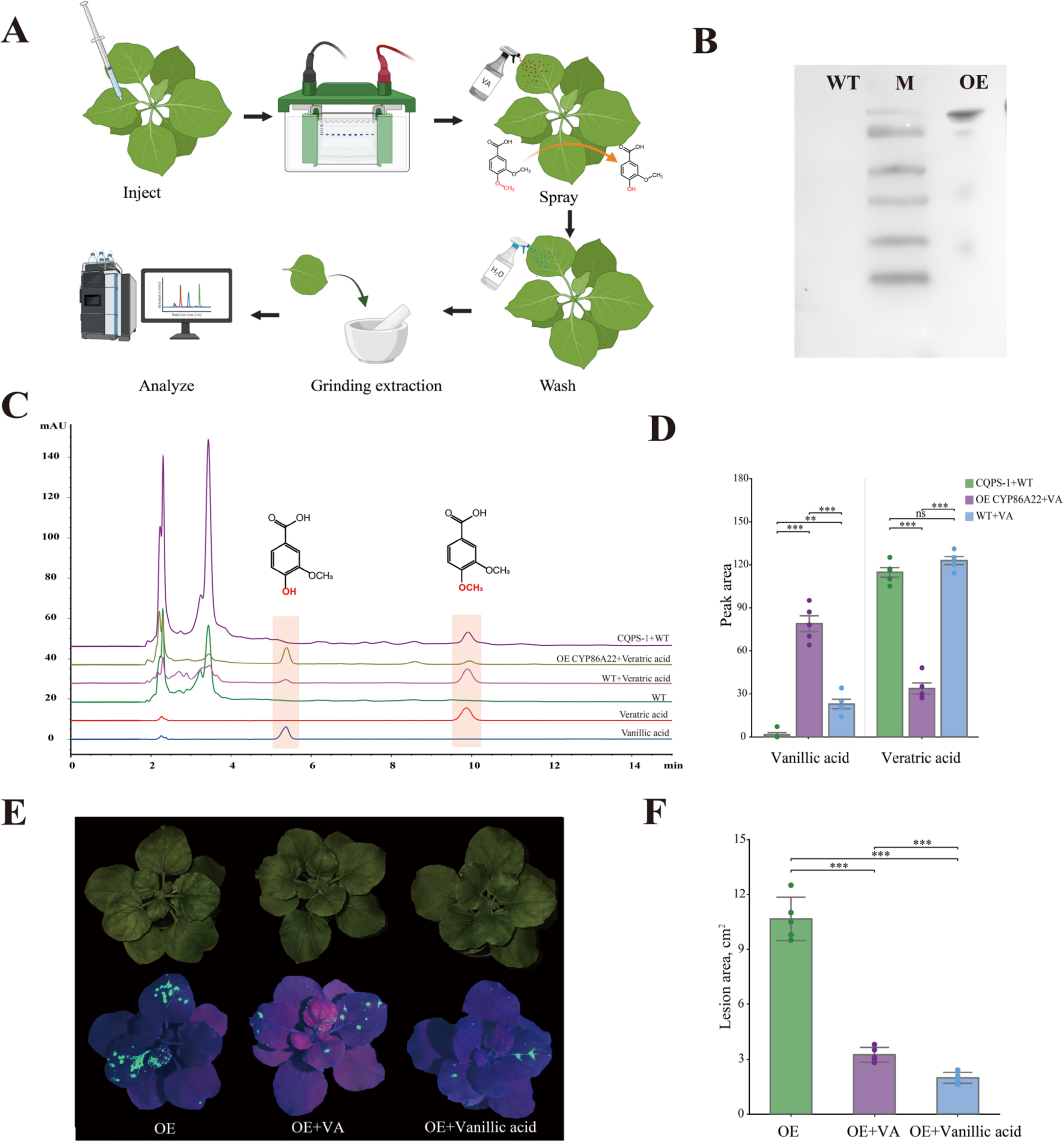
Transient overexpression of CYP86A22 mediates VA‐to‐VanA conversion and confers pathogen resistance in *Nicotiana benthamiana*. (A) Schematic diagram of the experimental procedure. (B) Western blot (WB) analysis confirming the accumulation of P450 protein in transiently transformed overexpression (OE) *N. benthamiana* lines, with no detection in wild‐type (WT) plants. (C, D) In vivo metabolic conversion in *N. benthamiana* analysed by high‐performance liquid chromatography (HPLC). Results show that (B) Western blot (WB) analysis confirming the accumulation of P450 protein in transiently transformed overexpression (OE) *N. benthamiana* efficiently converts externally applied veratric acid (VA) to vanillic acid (VanA), with VanA content in OE *N. benthamiana* being 3.46 times that in WT, while residual VA content in OE plants was only 27.4% of that in WT. WT plants showed low conversion capability, whereas this conversion ability was almost completely abolished in WT plants inoculated with *Ralstonia solanacearum* (CQPS‐1). (E, F) Analysis of vanillic acid‐mediated disease resistance. Before TMV inoculation, *N. benthamiana* were subjected to the following treatments: OE plants sprayed with veratric acid (OE + VA), or directly sprayed with vanillic acid (OE + VanA). Disease phenotype analysis revealed that the TMV lesion area in untreated OE plants was 10.66 cm^2^, which significantly decreased to 3.24 cm^2^ after VA application (OE + VA) and was further reduced to 1.98 cm^2^ after direct vanillic acid application (OE + VanA). These results demonstrate that the P450 enzyme enhances TMV resistance by converting VA to VanA, and that overexpression of P450 alone, in the absence of VA, does not confer TMV resistance.

To systematically identify the key enzyme responsible for metabolising VA, we first employed Microscale Thermophoresis (MST) to assess the direct binding affinity between the candidate P450s and VA. This screen identified CYP86A22 as the top binder, exhibiting a strong interaction with a dissociation constant (*K*_d_) of 2.289 μM (Figure 6E). We then verified the functional outcome of this binding by testing the in vitro catalytic activities of the candidates using HPLC. Consistent with the MST results, only CYP86A22 effectively converted VA into vanillic acid (VanA) (Figure 6F). This combined approach demonstrated that CYP86A22 is both the primary VA‐binding protein and the key catalyst for its conversion to VanA. To validate the function of CYP86A22 in plants, we performed *Agrobacterium*‐mediated transient overexpression of this gene in *N. benthamiana* leaves, with empty vector (WT)‐infiltrated plants serving as the negative control. Western blot analysis confirmed the successful accumulation of CYP86A22 protein in the overexpression (OE) samples, while it was undetectable in wild‐type (WT) plants (Figure 7B). Following exogenous application of VA, HPLC quantification showed that OE plants accumulated 3.46‐fold higher VanA levels and retained only 27.4% of the residual VA compared to WT plants (Figure 7C,D). These results demonstrate that transient overexpression of CYP86A22 is sufficient to efficiently convert exogenous VA into VanA in planta.

### 2.9 CYP86A22 Mediates *N. benthamiana* Disease Resistance Through Its Enzymatic Product VanA

To further investigate the physiological role of CYP86A22, we silenced its expression using virus‐induced gene silencing (VIGS). qRT‐PCR analysis confirmed efficient knockdown of *CYP86A22* transcripts (Figure S18). Under basal conditions (without VA treatment), silencing *CYP86A22* alone did not significantly alter susceptibility to *R. solanacearum* or TMV compared to control plants (Figures S11 and S12). This suggests that under normal growth conditions, other enzymes may compensate for the loss of CYP86A22, indicating functional redundancy in VA metabolism. Given that the enzyme's function is substrate‐dependent, we next investigated the biological significance of its enzymatic product, VanA. Wild‐type (WT) and CYP86A22‐overexpressing (OE) *N. benthamiana* were subjected to different treatments followed by TMV inoculation. Disease phenotype analysis revealed that untreated OE *N. benthamiana* itself was not resistant, with a lesion area (10.66 cm^2^) comparable to WT. However, after spraying with VA, the lesion area in OE *N. benthamiana* significantly decreased to 3.24 cm^2^. Notably, OE plants directly sprayed with VanA exhibited the strongest resistance, with the lesion area further reduced to 1.98 cm^2^ (Figure 7E,F). To determine whether VanA confers resistance by activating plant immunity, we examined the expression of defence‐related marker genes following exogenous VanA application. Quantitative RT‐PCR analysis revealed that VanA treatment did not significantly induce the expression of these genes compared to the mock control (Figure S13). Our further experiments confirmed that VanA does not activate the expression of defence‐related genes in *N. benthamiana*; instead, it controls TMV disease by directly inhibiting viral replication (Figure S14). These data clearly indicate that CYP86A22 against TMV in *N. benthamiana* by converting VA into VanA. For bacterial pathogen *R. solanacearum* , in vitro antimicrobial assays demonstrated that VanA exhibits stronger direct inhibitory activity than VA (Table S1). In the absence of the substrate VA, overexpression of the enzyme alone was insufficient to induce a resistance response.

To assess the broad‐spectrum antimicrobial activity of VanA, we performed in vitro antimicrobial assays against multiple plant pathogens, including the oomycete *Phytophthora* sp., the fungus *Fusarium oxysporum* and the bacterium *R. solanacearum* . At a concentration of 1 mg/mL, neither VanA nor VA showed obvious inhibitory effects on the mycelial growth of *Phytophthora* sp. or *F. oxysporum*, and no significant difference in antifungal activity was observed between VanA and VA (Figure S15). In contrast, VanA displayed stronger inhibitory activity against the bacterial pathogen *R. solanacearum* than the other tested compounds (Table S1). These results indicate that VanA functions as a selective antimicrobial metabolite with prominent inhibitory effects against the bacterial pathogen *R. solanacearum* .

## 3 Discussion

This study systematically deciphers a pivotal metabolic regulatory node in the tobacco– *R. solanacearum* interaction through multi‐omics and molecular biology approaches. We have, for the first time, unveiled an immune signalling pathway mediated by a specific P450 monooxygenase. This enzyme catalyses the conversion of VA—a susceptibility‐promoting phenolic acid induced by *R. solanacearum* infection—into VanA, which confers resistance against Tobacco Mosaic Virus (TMV) (Figure 7). Furthermore, we demonstrated that VanA exhibits a stronger inhibitory activity against *R. solanacearum* than VA. Importantly, our data show that VanA does not function as a classical defence‐activating signal; exogenous VanA application failed to induce the expression of plant defence‐related genes or trigger immune responses. Instead, VanA confers disease resistance through direct antimicrobial activity: it binds to TMV coat protein to interfere with virion assembly (Hu et al. 2022) and exhibits stronger direct bactericidal activity against *R. solanacearum* (Table S1). This discovery not only identifies a novel antimicrobial metabolite but, more importantly, elucidates a sophisticated plant counter‐strategy against pathogen ‘metabolic attack’: the enzymatic reprogramming of a ‘detrimental’ signal into a ‘beneficial’ one, thereby reversing the phenotype from susceptibility to resistance. This process perfectly exemplifies the intense ‘metabolic game’ between plants and pathogens.

Plants rely on secondary metabolites to build chemical defence systems, but their functions may be ‘hijacked’ by pathogens in specific interactions. VA, a phenolic acid widely present in plants (Montenegro et al. 2021), has derivatives with broad‐spectrum antibacterial activity (Narasimhan et al. 2009). In contrast to most studies reporting direct antibacterial effects of plant metabolites (Dang et al. 2023), our results clearly delineate the role of VA as a pathogen ‘collaborator’. It promotes disease not by directly enhancing pathogen growth but by multi‐targetedly suppressing the plant immune system (Figure 5). Firstly, VA significantly inhibits the flg22‐triggered reactive oxygen species (ROS) burst—a crucial early event in pattern‐triggered immunity (PTI) (Weralupitiya et al. 2024). Secondly, transcriptome analysis revealed that VA broadly and persistently downregulates the expression of a suite of key defence genes, including the NLR gene *NtTAO1*. This gene has been confirmed to activate downstream ROS signalling pathways by recognising TMV (Mestre and Baulcombe 2006). It also is a key disease‐resistant protein in *Arabidopsis thaliana* that responds to the pathogen effector AvrB, belonging to the TIR‐NB‐LRR family. It enhances disease resistance through synergistic interaction with RPM1. Abnormal function of this gene will lead to the interruption of defence signals and the decline of disease resistance (Eitas et al. 2008). Most importantly, dual‐luciferase reporter assays confirmed that VA directly inhibits the promoter activity of *NtTAO1*. During natural infection, the accumulation of VA precedes the downregulation of *NtTAO1*, strongly supporting its causal role as an immune‐suppressive signal in vivo (Figure 5F). To further explore the mechanism underlying VA‐mediated immune suppression, we quantified the endogenous levels of SA and JA in *N. benthamiana* following VA treatment (Figure S16). The results revealed that exogenous VA application significantly reduced the accumulation of SA, whereas the content of JA remained largely unchanged. Consistently, the expression patterns of the corresponding defence‐related genes showed no obvious differences between gene‐silenced and non‐silenced *N. benthamiana* plants.

Confronted with the immune‐suppressive assault of VA, we postulated that plants must have evolved corresponding detoxification or countermeasure mechanisms. This hypothesis is grounded in solid literature evidence. Firstly, studies in microbial systems have reported that P450 enzymes can catalyse the demethylation or hydroxylation of VA, yielding VanA (Bell et al. 2012; Podgorski et al. 2022). Secondly, and more critically, independent biochemical research has confirmed that VanA can directly bind to TMV coat protein, interfering with virion assembly or stability, thereby effectively inhibiting its infection and replication (Hu et al. 2022). These two independent lines of evidence form a compelling logical loop: a known enzymatic reaction (CYP86A22‐catalysed VA to VanA) could produce a known functional antimicrobial product (VanA inhibiting TMV). This led us to propose the plausible conjecture that plants might employ a similar P450‐mediated reaction to execute a ‘metabolic counterattack’ against VA.

Our functional genomics and biochemical experiments successfully validated this conjecture. Notably, the disease resistance observed here does not involve the activation of the plant's own inhibitory capacity against TMV; instead, it arises from the direct inhibitory effect of metabolites on TMV. We identified CYP86A22 from multiple candidates as the specific executor of this conversion reaction (Figure 7). Its high substrate binding affinity, catalytic specificity in vitro, and ability to drive the efficient conversion of VA to VanA in transient overexpression assays collectively confirm its core physiological function. Consequently, the role of CYP86A22 transcends simple detoxification; it acts as an immune signal converter, transforming a ‘disease‐promoting’ metabolic state into a ‘defence‐activating’ one.

However, a fundamental question arises: what is the origin of the VA that accumulates substantially in plants upon pathogen infection? Currently, its biosynthetic pathway in plants remains unknown. Is VA a by‐product of aberrant metabolic reprogramming in plants under stress, or is it an effector molecule actively secreted by *R. solanacearum* to manipulate host immunity? To address this question, we performed in vitro culture experiments. *R. solanacearum* was cultured in two media: *N. tabacum* extract medium and the commonly used B medium. After 72 h of incubation, VA and VanA were not detected in either of the bacterial culture supernatants (Figure S19). These results clearly demonstrate that VA is not a metabolite directly secreted by the pathogen, but rather a host‐derived metabolite whose accumulation is induced and enhanced via pathogen‐mediated manipulation of host metabolic pathways. Clarifying the biosynthetic origin of VA—whether host‐ or pathogen‐derived—is crucial for a complete understanding of this interaction model. Although we speculate it originates from host metabolism, this remains a key direction for our future research.

Through in‐depth investigation of *NtTAO1*, we revealed its complex role in the defence network and adeptly resolved the phenotypic paradox between the in vitro antibacterial effect and the in planta disease‐promoting effect of VA.

### 3.1 Core Role Against TMV

Silencing *NtTAO1* drastically enhanced susceptibility to TMV, and exogenous VA application could not further exacerbate the disease. This clearly indicates that *NtTAO1* is a non‐redundant, key defence component against TMV in *N. benthamiana* and is a primary downstream target for VA‐mediated immune suppression (Mestre and Baulcombe 2006).

### 3.2 Indirect Role Against *R. solanacearum*

In contrast, silencing *NtTAO1* alone did not significantly alter the progression of bacterial wilt caused by *R. solanacearum* , suggesting the pathogen may possess effectors that compensate for or circumvent the immunity mediated by this gene (Tsuda et al. 2009). However, applying VA to silenced plants significantly accelerated disease development. This reveals a deeper mechanism: during *R. solanacearum* infection, VA likely functions by suppressing a broader NLR gene network that includes *NtTAO1*. When this network is already partially disrupted by VA, removing one of its key nodes (*NtTAO1*) produces a synthetic effect, leading to a comprehensive collapse of the immune system facing multiple pathogen attacks.

Based on these findings, we propose a working model for the plant–pathogen metabolic game: *R. solanacearum* infection (potentially via secretion or other means) leads to VA accumulation in tobacco, which subsequently suppresses the immune network involving nodes like *NtTAO1*. Concurrently, the plant activates a ‘metabolic counterattack’ mediated by CYP86A22, converting VA into VanA, which directly acts against pathogens such as TMV and restores disease resistance. Consistent with this, silencing CYP86A22 alone did not compromise basal resistance (Figure S11), suggesting that under non‐stress conditions, redundant enzymes may contribute to VA turnover. However, the ability of CYP86A22 overexpression to drive efficient VA‐to‐VanA conversion and enhance resistance upon VA application (Figure 7) demonstrates that this enzyme is sufficient to execute the metabolic counterattack when VA levels are elevated, such as during pathogen infection. Thus, while other enzymes may participate in VA metabolism, our gain‐of‐function and in vitro data establish CYP86A22 as a key player in this defence strategy.

*R. solanacearum* served as the initial model pathogen to uncover the VA‐mediated immunosuppression and the metabolic counterattack mechanism. However, TMV was employed to functionally validate the antiviral activity of VanA, because VanA has been previously reported to directly bind the TMV coat protein and inhibit viral assembly (Hu et al. 2022), providing a well‐defined and quantifiable readout for its defensive function. Notably, the enhanced resistance to *R. solanacearum* observed upon sole overexpression of *CYP86A22* stems from its key enzymatic function: during *R. solanacearum* infection, the pathogen induces VA accumulation in plants, and CYP86A22 specifically catalyses the conversion of VA to VanA. In contrast, VanA's effects on *R. solanacearum* are primarily attributed to its direct antibacterial activity (Table S1) and its role in alleviating VA‐mediated immune suppression—processes that involve more complex plant–pathogen interactions. Therefore, TMV offers a more straightforward system to demonstrate the gain‐of‐function conferred by CYP86A22‐mediated VA‐to‐VanA conversion, while the bacterial pathosystem remains essential for understanding the ecological and metabolic context of this immune strategy.

It is worth noting that this intricate metabolic interplay between plant and pathogen occurs within the dynamic physicochemical context of the rhizosphere. Changes in soil pH, for instance, are not merely a background condition but a consequence of the metabolic activities of both partners. Root exudates, including phenolic acids like VA, are known contributors to soil acidification (e.g., in continuous cropping systems) (Ma et al. 2022). This alteration in pH, in turn, can profoundly reshape the metabolic profiles of both the plant and the rhizosphere microbial community, forming a complex feedback loop (Hu et al. 2018). Integrating this rhizosphere perspective will be essential for a holistic understanding of how the identified ‘metabolic counterattack’ functions in a natural, complex soil environment. However, it should be noted that direct evidence linking soil acidification to the transcriptional activation of *CYP86A22* is currently lacking (Figure S17), and this represents an important avenue for future investigation.

The theoretical significance of this study lies in expanding the understanding of plant immunity from the level of protein interactions to the dimension of dynamic metabolic regulation. The precise modification of phenolic acid signals by P450 enzymes represents a novel immune fine‐tuning mechanism. Looking forward, several key questions warrant exploration: What is the specific downstream signalling pathway activated by VanA? How is the expression of the CYP86A22 enzyme itself precisely regulated by immune signals? Furthermore, while our current data do not support a direct link between soil acidification and the transcriptional activation of *CYP86A22* (Figure S17), investigating whether such rhizosphere physicochemical factors can modulate the efficiency or specificity of this metabolic conversion represents an intriguing avenue for future research. Is this pathway conserved across different crop‐pathogen systems? Addressing these questions holds significant theoretical value and provides promising new avenues for controlling crop diseases through metabolic engineering breeding (manipulating CYP86A22 expression) or developing green immune‐priming agents based on VanA.

## 4 Materials and Methods

### 4.1 Plant Materials, Pathogens and Basic Disease Analysis

#### 4.1.1 Plant Materials and Growth Conditions

This study utilised tobacco cultivars *N. tabacum* cv. ‘K326’, and *N. benthamiana*. All plants were cultivated in controlled environment growth chambers under a 12‐h light/12‐h dark photoperiod, with day/night temperatures set at 30°C/24°C and relative humidity maintained at 60%–70%.

#### 4.1.2 Pathogens and Inoculum Preparation

The bacterial strains *R. solanacearum* CQPS‐1 and *P. syringae* pv. *tabaci*, as well as Tobacco Mosaic Virus expressing green fluorescent protein (TMV‐GFP), were kindly provided by the College of Plant Protection, Southwest University. *R. solanacearum* and *P. syringae* pv. *tabaci* were cultured in B liquid medium at 28°C with shaking at 200 rpm until the late logarithmic growth phase (OD_600_≈0.8). Bacterial cells were harvested and resuspended in sterile deionised water to the desired concentrations for inoculation ( *R. solanacearum* for root irrigation: 10^7^ CFU/mL; *P. syringae* pv. *tabaci* for needle infiltration: 10^6^ CFU/mL). TMV‐GFP inoculum was prepared by homogenising 0.1 g of systemically infected leaf tissue in 2 mL of phosphate‐buffered saline (PBS, pH 7.4), followed by centrifugation at 3000 × *g* for 5 min to collect the supernatant (Wei et al. 2025).

#### 4.1.3 Disease Severity Assessment

The severity of bacterial diseases was evaluated at specified dpi by measuring lesion area or using standardised disease indices. The severity of TMV infection was assessed at 7 dpi by quantifying the lesion area on leaves. All treatments included at least five plants as biological replicates, and the entire experiment was independently repeated three times.

### 4.2 Soil and Plant Tissue pH Measurement

#### 4.2.1 Rhizosphere Soil pH Measurement

Paired rhizosphere soil samples from *N. tabacum* plants exhibiting clear bacterial wilt symptoms and adjacent healthy plants were collected in August from multiple *N. tabacum* ‐growing regions in Guizhou Province. Samples were air‐dried, visible debris was removed, and soils were passed through a 2‐mm sieve. Precisely 10.0 g (dry weight) of soil was mixed with 50 mL of deionised water (1:5, w/v). The suspension was vigorously shaken for 30 s and allowed to settle for 30 min before filtration through qualitative filter paper. The pH of the supernatant was measured using a Mettler Toledo FE28 pH meter calibrated with standard buffers (pH 4.0, 7.0 and 10.0). Each sample was measured in triplicate.

#### 4.2.2 Plant Tissue pH Extraction and Measurement

Diseased tissue from *N. tabacum* cv. K326 plants showing approximately 70% wilting at 5 days post‐inoculation with *R. solanacearum* (10^7^ CFU/mL), along with healthy control tissue, was harvested. One portion of fresh tissue was mixed with deionised water at a 1:5 (w/v) ratio, sonicated in an ice bath (40 kHz, 10 min), and the supernatant was used for pH measurement. Another portion of tissue was freeze‐dried, subsequently rehydrated at a 1:10 (w/v) ratio with deionised water, and similarly subjected to sonication and filtration before pH measurement of the filtrate.

### 4.3 Metabolomic Analysis and Apoplastic Fluid Cultivation

#### 4.3.1 Apoplastic Fluid Collection and Dynamic pH Monitoring

Leaves from healthy six‐leaf‐stage *N. tabacum* K326 were cut into 1 cm^2^ explants. Approximately 100 g of tissue was subjected to cyclic negative‐pressure infiltration (−0.1 MPa, 5 min per cycle, repeated 5 times) using a syringe containing 200 mL of ultrapure water to collect apoplastic fluid. The collected fluid was filter‐sterilised through a 0.22‐μm membrane and inoculated with 5% (v/v) of a pre‐activated *R. solanacearum* suspension (initial OD_600_ = 0.2). The culture was incubated at 28°C with shaking at 220 rpm. Samples were collected aseptically at 24, 48 and 72 hpi, allowed to stand for 5 min, and the pH was measured.

### 4.4 Functional Validation of Acidic Metabolites

#### 4.4.1 Effect on *R. solanacearum* Pathogenicity

Six organic acids showing significant abundance changes between diseased and healthy tissues in the metabolomics data were selected. Chromatography‐grade compounds were dissolved in methanol to prepare 10 mg/mL stock solutions, which were filter‐sterilised through a 0.22‐μm nylon membrane and diluted with ultrapure water to working concentrations of 0.5 and 1.0 mg/mL (final methanol concentration < 2%). Seven‐leaf‐stage ‘K326’ seedlings were uniformly sprayed with these solutions (1.0 mL/plant). Twelve hours post‐treatment, plants were challenge‐inoculated via quantitative root irrigation with a *R. solanacearum* suspension (10^7^ CFU/mL, 10 mL/plant). Plants sprayed with 2% methanol and uninoculated plants served as the solvent control and blank control, respectively. Disease symptoms were monitored and recorded daily for 14 days, and disease indices were calculated.

### 4.5 P450 Enzyme Functional Verification

The three‐dimensional structure of the target P450 enzyme was predicted using AlphaFold2 (Bryant et al. 2022), target P450 protein sequence was obtained from the https://solgenomics.net/organism/Nicotiana_tabacum/genome database. Molecular docking of the enzyme with veratric acid (VA) was performed with AutoDock 4.2 (Forli et al. 2016), and the results were visualised using PyMOL2.5 (http://www.pymol.org). Binding affinity and catalytic plausibility were evaluated by analysing the binding free energy and interaction modes with the heme cofactor and key amino acid residues. To further investigate the dynamic stability and binding mechanism, a 100 ns molecular dynamics (MD) simulation was conducted using GROMACS. The binding free energy in a solvent environment was calculated from the equilibrated trajectory using the MM/PBSA method (Valdes‐Tresanco et al. 2021).

#### 4.5.1 In Vitro Catalytic Activity Assay

Four candidate P450 recombinant proteins were purified. In the reaction system, purified P450 protein (final concentration 5 μM) was incubated with the substrate VA (final concentration 1 mM) and the cofactor NADPH (final concentration 2 mM) at 37°C for 2 h. After the reaction, the products were analysed by HPLC under the following conditions: C18 column; mobile phase: methanol and 0.1% formic acid in water, using a gradient elution programme; detection wavelength: 280 nm. Product identification and quantification were performed by comparing retention times and UV spectra with those of a vanillic acid (VanA) standard.

#### 4.5.2 Protein‐Substrate Interaction Analysis

The purified CYP86A22 protein was fluorescently labelled using the Monolith RED‐NHS 2nd Generation labelling kit. The labelled protein was mixed with a series of concentrations of VA. The catalytically inactive P450 1# protein served as a negative control. Changes in microscale thermophoresis were measured, and the dissociation constant (*K*_d_) was calculated using the instrument's software (Wang et al. 2024).

#### 4.5.3 Generation of Transgenic *N. benthamiana* and In Planta Metabolic Validation

The gene encoding the CYP86A22 enzyme was cloned into the plant overexpression vector pCAMBIA1302 and introduced into *N. benthamiana* via *Agrobacterium*‐mediated leaf disc transformation (Lei et al. 2025). Positive transgenic seedlings were selected by hygromycin resistance and confirmed by PCR. Further confirmation of target protein accumulation in overexpression (OE) lines was performed by Western blot analysis using a GST‐tag specific antibody. To validate in vivo function, leaves of wild‐type (WT) and OE line *N. benthamiana* were sprayed with a 0.5 mg/mL VA solution (1 mL/plant). Samples were collected 72 h post‐treatment, ground in liquid nitrogen, and metabolites were extracted with methanol at a ratio of 10 mL/g of plant material. The extracts were concentrated and filtered through a 0.22‐μm membrane before HPLC analysis to quantitatively compare VA consumption and VanA production.

#### 4.5.4 VanA‐Mediated Disease Resistance Assessment

Wild‐type and P450‐overexpressing *N. benthamiana* were subjected to three treatments: (1) mock control (no treatment); (2) foliar spray with 1 mg/mL VA; (3) direct foliar spray with 1 mg/mL VanA. All treatments were applied 72 h prior to inoculation with TMV‐GFP. At 72 h post‐inoculation, the lesion area on leaves was measured and statistically analysed to assess disease severity for each treatment. The amino acid sequence of the P450 protein can be found in the Supporting Information.

### 4.6 Effects of VA on Plant Immunity and Cellular Structure

#### 4.6.1 Transcriptome Sequencing and Analysis

Six‐leaf‐stage *N. tabacum* cv. K326 plants were sprayed with a 1 mg/mL VA solution. Leaf tissues were collected at 2, 4, 8, 16 and 32 h post‐treatment (hpt) and flash‐frozen in liquid nitrogen. Total RNA was extracted using the TRIzol method. RNA quality and integrity were assessed using a Nanodrop 2000 spectrophotometer and an Agilent 5300 Fragment Analyzer. Qualified samples were used for library construction, followed by paired‐end 150 bp sequencing on an Illumina NovaSeq X Plus platform. Raw sequencing data were aligned to the *N. tabacum* cv. K326 reference genome (*SGN*, https://solgenomics.net/datasets/genome_assemblies/tobacco/; accessed 2025‐05‐23). Fuzzy clustering (Mfuzz) and functional enrichment analysis were performed on differentially expressed genes. Heat maps, bubble charts and Sankey diagrams were constructed using R language (4.2.2) packages ggplot2 and ggalluvia. Fuzzy clustering analysis of plant disease‐related genes (partitioned into eight clusters) was conducted based on the Mfuzz method, utilising online tools OmicShare (https://www.omicshare.com/tools) (Mu et al. 2024). This analysis aimed to identify co‐expressed gene modules associated with plant disease responses, with clustering results visualised through heat maps to show expression patterns, bubble plots to highlight enriched functional categories, and Sankey diagrams to illustrate relationships between gene clusters and disease‐related pathways.

ROS production was measured using a luminol‐based chemiluminescence assay, as previously described. Leaf discs (4 mm diameter) from 4‐week‐old *N. benthamiana* were floated overnight in water. The next day, water was replaced with a reaction solution containing 20 μg/mL horseradish peroxidase (HRP), 100 μM luminol and 1 μM flg22. Luminescence was immediately recorded every 2 min for 60 min using a multimode plate reader. For VA treatment, plants were sprayed with 1 mg/mL VA (in 1% methanol) 2 h prior to leaf excision.

For visual ROS detection, DAB staining was performed as described previously. Leaves were vacuum‐infiltrated with 1 mg/mL DAB solution (pH 3.8), incubated in the dark for 12 h, and then bleached with 95% ethanol (Zhao et al. 2025).

#### 4.6.2 Cellular Ultrastructure Observation

Leaves of *N. benthamiana* sprayed with 1 mg/mL VA for 12 h were sampled. Tissue was rapidly cut into 3–5 mm^2^ pieces and immediately fixed in 2.5% (v/v) glutaraldehyde in 0.1 M phosphate buffer (pH 7.4) at 4°C for 24 h. After rinsing with 0.1 M phosphate buffer, samples were post‐fixed with 1% (w/v) osmium tetroxide for 2 h. Dehydration was performed using a graded ethanol series, followed by acetone substitution. Tissues were infiltrated and embedded in Epon 812 epoxy resin and polymerised at 60°C for 48 h. Ultrathin sections (50–70 μm) were prepared using a Leica EM UC7 ultramicrotome, stained with uranyl acetate and lead citrate, and observed under a JEOL JEM‐1400 transmission electron microscope for imaging.

### 4.7 Functional Studies of *NtTAO1*

#### 4.7.1 Virus‐Induced Gene Silencing (VIGS) (Fernandez‐Pozo et al. 2015)

A specific fragment of the target *NtTAO1* was cloned into the Tobacco Rattle Virus (TRV)‐based VIGS vector TRV2. The recombinant plasmid (TRV2‐*NtTAO1*), a positive control (TRV2‐PDS, targeting the phytoene desaturase gene which induces photobleaching) and an empty vector control (TRV2) were separately electroporated into *Agrobacterium tumefaciens* strain GV3101 harbouring the helper plasmid TRV1. Cultures of *Agrobacterium* containing TRV1 and the respective TRV2 constructs were adjusted to OD_600_ = 1.0, mixed in a 1:1 ratio and infiltrated into leaves of six‐leaf‐stage *N. benthamiana* plants. After the positive control (TRV2‐PDS) plants showed systemic photobleaching (approximately 12 days post‐infiltration), the silencing efficiency of *NtTAO1* in TRV2‐*NtTAO1* plants was verified by RT‐qPCR. Successfully silenced plants were used for subsequent functional assays.

#### 4.7.2 Dual‐Luciferase Reporter Assay

The promoter region of *NtTAO1* (1779 bp upstream of the transcription start site) was predicted using TBtools software and cloned into the dual‐luciferase reporter vector pGreenII 0800‐LUC (Chen et al. 2023). The recombinant plasmid was transformed into *A. tumefaciens* GV3101 containing the pSoup19 helper plasmid. A single colony was cultured to an OD_600_ of 0.6–0.8, and the bacterial cells were collected and resuspended in infiltration buffer. The bacterial suspension was injected into the abaxial side of leaves of 4‐week‐old *N. benthamiana* plants. Twelve hours post‐infiltration, leaves were sprayed with a 1 mg/mL VA solution and incubated for an additional 8 h. The entire leaf was then sprayed with luciferase substrate solution, and luminescence signals were captured and analysed using a plant in vivo imaging system.

## Funding

This work was supported by National Key Research and Development Program of China (2022YFD1700300) and National Natural Science Foundation of China (32502535, 32202398 and 32202338).

## Conflicts of Interest

The authors declare no conflicts of interest.

## Supporting information


**Figure S1:** Metabolomic differences between diseased and non‐diseased tissue.
**Figure S2:** qRT‐PCR validation of RNA‐seq data at 4 h.
**Figure S3:** Differential expression analysis of genes after VA treatment.
**Figure S4:** GO enrichment analysis of DEGs after VA treatment.
**Figure S5:** Phylogenetic tree of NtTAO1 and its homologues.
**Figure S6:** Heat map of P450‐related gene expression.
**Figure S7:** Molecular docking diagram of P450 protein 1 with VA.
**Figure S8:** Molecular docking diagram of P450 protein 2 with VA.
**Figure S9:** Molecular docking diagram of P450 protein 3 with VA.
**Figure S10:** Molecular docking diagram of P450 protein 4 with VA.
**Figure S11:** Pathogenicity assay of CYP86A22‐silenced and overexpressing plants.
**Figure S12:** Defence‐related gene expression in CYP86A22‐overexpressing plants.
**Figure S13:** Defence‐related gene expression under VA and VanA treatments.
**Figure S14:** TMV accumulation after VanA treatment.
**Figure S15:** Antifungal activity assay of VA and VanA.
**Figure S16:** SA and JA contents after VA treatment.
**Figure S17:** CYP86A22 expression under acidified soil condition.
**Figure S18:** VIGS silencing efficiency of CYP86A22.
**Figure S19:** HPLC analysis of VA and VanA in *Ralstonia solanacearum* culture supernatants.
**Table S1:** EC50 of tested compounds against *Ralstonia solanacearum* .
**Table S2:** Primers used in this study.
**Table S3:** Molecular docking binding energies.
**Data S1:** Sequences.
**Data S2:** Gene sequence of NtG28897 (NtTAO1).
**Data S3:** Amino acid sequence of CYP86A22.

## Data Availability

The data that support the findings of this study are available in the Supporting Information of this article.
