# Supplementary material for: Metabolic Reprogramming of a Phenolic Acid by a Plant P450 Monooxygenase Reverses Bacterial Immunosuppression
Source: Plant Biotechnol J. 2026 Mar 31;24(7):4672–88. doi: 10.1111/pbi.70660 (PMC13278537; doi:10.1111/pbi.70660)
Supplement: Supplementary file 1 — Figure S1: Metabolomic differences between diseased and non‐diseased tissue. Figure S2: qRT‐PCR validation of RNA‐seq data at 4 h. Figure S3: Differential expression analysis of genes after VA treatment. Figure S4: GO enrichment analysis of DEGs after VA treatment. Figure S5: Phylogenetic tree of NtTAO1 and its homologues. Figure S6: Heat map of P450‐related gene expression. Figure S7: Molecular docking diagram of P450 protein 1 with VA. Figure S8: Molecular docking diagram of P450 protein 2 with VA. Figure S9: Molecular docking diagram of P450 protein 3 with VA. Figure S10: Molecular docking diagram of P450 protein 4 with VA. Figure S11: Pathogenicity assay of CYP86A22‐silenced and overexpressing plants. Figure S12: Defence‐related gene expression in CYP86A22‐overexpressing plants. Figure S13: Defence‐related gene expression under VA and VanA treatments. Figure S14: TMV accumulation after VanA treatment. Figure S15: Antifungal activity assay of VA and VanA. Figure S16: SA and JA contents after VA treatment. Figure S17: CYP86A22 expression under acidified soil condition. Figure S18: VIGS silencing efficiency of CYP86A22. Figure S19: HPLC analysis of VA and VanA in Ralstonia solanacearum culture supernatants. Table S1: EC50 of tested compounds against Ralstonia solanacearum . Table S2: Primers used in this study. Table S3: Molecular docking binding energies. Data S1: Sequences. Data S2: Gene sequence of NtG28897 (NtTAO1). Data S3: Amino acid sequence of CYP86A22. [file PBI-24-4672-s001.docx]

**Metabolic Reprogramming of a Phenolic Acid by a Plant P450 Monooxygenase Reverses Bacterial Immunosuppression**

Hua Wang^1^, Xinrui Wang^1^, Xiaoshuang Peng^1^, Ruiyu Yang^1^, Tao Liu^1^, Huidi Liu^1^, Chao Zhao^2^, Hong Zhou^1^, Liang Yang^1^, Shili Li^1^*, Wei Ding^1^*

1. Laboratory of Natural Products Pesticides, College of Plant Protection, Southwest University, Chongqing 400715, China
2. Biotechnology Laboratory, Shijiazhuang Institute of Pomology, Hebei Academy of Agriculture and Forestry Sciences, Shijiazhuang 05000, China;

*Corresponding author: Wei Ding, E-mail: [dingw@swu.edu.cn](mailto:dingw@swu.edu.cn), Shili Li E-mail: yuanls2016@swu.edu.cn

>gene_28897 - Up_Stream_Len 1799

ttcatgacaagttcacttgaaacagtatataatatctctcggaaatttcatgatagtaaaatatgccagctgaagtatacagaaatgaaatcaagtgcataatctcagagccacagtcacttagtcctcccatttactccaacctcacagtcgctcggcactcgcgctcatcactctcactcgtcactcgtcacttgtcattcgcactcagggggtacccgcgctcacttgggtgtgtatagactccggaggggatccttcagcccaagcgctataacaagccaatcatggcatagatcaataaaatatgttgcggcatgcaacccgatcccaattatatcctcacaatcaggccctcggcctcacttagtcatcaatatctctagtctctcgggccctcaataatcagcgtaaacaacgatgatataatgcatcaataaagaacaatagagactgatatataatatgcaagtaaaattgcgactgagtacaaaataacaatttagcagataattcaacatgtaacacaacccctgtgggtccctacagtaccaacacatatcctaaccataatttctaacatgacttgcattttaattttttataacacatggagaatatacggatatcaacagagtattcaactatatagttccattgaattgaccaagtcgaaattcctacagtgcacgcccgtcatctagcatgtgtgtcacctcaacaccaatcacatgacacataatacgggatttcataccctcaaaactaaatttagaattgttacttaccttaaatcggacaaatctctactccaacacacctttgcctcgctaaacagcctctgaatgctttgaatctagccacaaataattccatacaatcaatacgagctaaagggatcaattccataagaaaatactaagcacttaatgaaaagtcaaaaagtcaactcaaaagtaggcccccgggcccacgtctcggaatcggataaaagttgtcacacctcctttttcccgaagacatagggagttttttcaattaaagtgatattgttcgaaatgagattatttatttaattcagagtcgccacttggaataatttatggtgtcccaagctggttgacctcttgtaggacttgctgtataattttttcaataaactttgattcatgcctacaaatgttttagcaaaaacaaaaaaattaaaagaaatactactccagtaaataaaactttgatatttgaaaatatttgacgacatatcaacatcaatagccaagacataacacagagggatgaatctaagcaacaaaatagataacttactgcgtcatataggatattttaagaaataattaagttatatatattgatagtgtaaatatttcaataaaatgtcaatgtataaaacttaaaactctttttaacagttagtatcataccgtataaacaattgaacaaataacaaatgaaattatatagttgtatcaagaaagtatacccgtcagcaacattttgcaaatcccatccagataaatttgcagcttcagtaagtgcagctgtccatttctgcaccatttgagccccaattgatcgttccttatgttttgccaaagcttccccaaataacccagtttgctttcgcacttgagaaggatcaacatcatagaaaataggcaaaaccatctgctttaatttctctttgcattcaagaatcttaactagttcatttagacaccaactggaggaagcataatttcttgagaaaactacaatggaaattcttgatccttcaattg

>mRNA_49801 gene_28897 id=AT5G36930.1: evalue=2e-46: annot= Disease resistance protein (TIR-NBS-LRR class) family; id=Solyc09g092410.2.1:evalue=0.0:annot='Tir-nbs-lrr, resistance protein

gaatttaaaagaatccaaaaaaatggtaatccccttcaaaagttttctggtcagtacagattgatctttctactaaaaagtatgagattagtcttcggtcatattttgatacatttgatgaacgcgcgaagtgcggacaattatactagtgtaatataaaatacaacttgattagctctaaaggaaagttatcttgactcagaacgttgctcttttacaaggaggagttgaaactctttttgggtacttagaataagaatgatccacgtctcccagcaaagatacaactttaccatgttggtctaaatataacagatggatccctatcttctttattgtaatgttgtcagcccacacttctatcatttccccgcctttgatcatctggccatctaaagctttatgtaagtaagatatgtaatatacacatgacagaggttttttcccttttctccaccgaatatttgaaggctttccatgtgcaatactaatcctgaagcatggattatgcgggactacatcgaacaagtcggaaacaaaccagagaaccattcctaagaagttattatgtgttggcatagtcaaacacatagatgaagctgttacttgattcctgcaccattctggaatctcattacattcgagataaattctaatagtatcctgcatcatatatcgagcaggtgcactaaatgcaaatagagcaggtgcactaaagaagccttcattgaatggattctgcagagaactacaattaatcatattaatccgctctatagaagggaggttgtccaactctgtaatcttgaccaatttttggcaattataaaggttaattttataaagattctctaaatttgatactgacgggagtgtttgaagattctcacagtcattcaaacacaacttctccaacaatcgtaacttagaaaaatcaaagggtagacaatagaaactgttgccactcaaatccaaatattttaaggaggacaagctcccaatatccctaggaatatcagcctcggacaaattacagtatgtaaggctcaaatcgcatacaaaagttggcaaggaatattgtatctgatggactcctcttccagaaatactccttttggcctctaacttttgacctcccactatcaaagttacaagatttcttagcatttcaacagatctaggaaattgttttatacccgtacgagatgcataaagagatcttagacttttcatatctccaaggtcatctggcagtgtttttataaatgagcagttactaatgctcaagtaattaacggatattagctggcatatgctgcttggaagatccgtaagtttttcgcaaccagacatagatagttcaattagtctggacaaatttcctattgatggatggatctcagtcagacttgagcaaccatggagatacaaagtctcaagactcagtgaaccattgaagtttggagtgcttctgagttgcttgcaatgggagagattcagctccttcaaacttctacaacactgcgtattcaaaccaaactcttggatatcactctcccgcatatctagaactacaagtttgtcagctggaaaatttgatggtatatattttaaaggacattttttccaagacaaccatctgagcttcttggacaacaacccaaaatctccactaatatgtaactcatccattataagaaccctaaggnnnnnnnnnnnnnnnnnnnnnnnnnnnnnnnnnnnnnnnnnnnnnnnnnnnnnnnnnnnnnnnnnnnnnnnnnnnnnnnnnnnnnnnnnnnnnnnnnnnngcatgtggctttcatgctaaaagtgcaattgcaactttagtccaaaaacacttgctccaaagaattgggtatcatttggtgatgcatgatctagtgcgagatatgggaagagaaatcgttcgtatggaatcatctcgagaccctggaaaacggagtagattgttcatccctcaagaagttcgtgatgttctacaaggaaatgaagttagtaaatccttcatctttatcttggttatatatttttttcaattaaattgtttcttgcttataatttttgttgccataataagaattcatttgtgcatcgtctctggttagtaagtattttgtagttgtttttcaaccattgtaaattatgacatttgctaaactctagatgcaatttgttctctttattcatatattttttttttcctttgatgttatcatgtgaacttattgaatgatgctagacagtttcgaaaggctctcatcttagcttttttgagaaattttacatttgtttaacatcctctaatgtgtaagttcatttttgttgatacatggtgtattctctttttttgtccaccaatttgtataaatcatttttctctcttaatttctgggacctaacatgagcttaagttacttttctttttactgagctgcagggttccgaaaatgtagaagtgctgaaggtagatcgagggacattaaagggagtgaacttgagcaccaaagcatttgagcaaatgaaaaaccttagggttcttataatggatgagtt

**Amino Acid Sequence of P450 Protein**

>rf 1 sample sequence

MDIAIALLLFTAITCYLLWFTFISRSLKGPRVWPLLGSLPGLIENSERMHDWIVDNLRACGGTYQTCICAIPFLARKQGLVTVTCDPKNLEHILKTRFENYPKGPTWQAVFHELLGQGIFNSDGDTWLFQRKTAALEFTTRTLRQAMARWVNRAIQLRFCPILKTAQLESKPVDLQDLLLRLTFDNICGLAFGKDPQTLAPGLPENTFSSAFDRATEASLQRFILPEVIWKLKKWLGLGMEVSLNRSLVQLDKYMSDIINTRKLELMSQQKDGNPHDDLLSRFMKKKESYTDKFLQHVALNFILAGRDTSSVALSWFFWLVIQNPVVEQKILHEICTVLIETRGSDTSSWLDEPLAFEEVDRLTYLKAALSETLRLYPSVPEDSKHVVVDDLLPDGTFVPAGSSITYSIYSAGRMKTTWGEDCLEFKPERWLTPDGKKFVMHEQYKFVAFNAGPRICLGKDLAYLQMKSVAAAVLLRHRLMVAPGHKVEQKMSLTLFMKDGLKVNLLPRDLTLLTDSLKKEKEVQLVQKLHRDKDE*

>rf 1 sample sequence 2

MMIIAIVAAYLLWFKSITKSMKGPKGPKMWPIVGSLPGLLENGTRMHEWIAENLRACTGTYQTCIFAIPFLARKQGLVTVTCDPKNLEHILKVRFDNYPKGPTWQAVFHDLLGEGIFNSDGDTWLFQRKTAALEFTTRTLRQAMGRWVNRAIKNRFCPILEMAQVQDKPVDLQDLLLRLTFDNICGLAFGKDPETLSPELPENNFAMSFDRATEASLHRFIMPEFVWKLKKMLGLGMEVSLSHSLKQVDDYMTNVINTRKLELLNHQNGGPQHDDLLSRFMKKKESYSDKFLQHVALNFILAGRDTSSVALSWFFWLVSLNPRVEEKILVELCTVLAETRGNDTSKWLEEPLVFEEVDRLTYLKAALSETLRLYPSVPEDSKHVICDDYLPDGTFVPAGSNITYSIYSTGRMKFIWGEDC

LEFKPERWMSQDGNKYQVQDAFRFVAFNAGPRICLGKDLAYLQMKSIVAAVLLRHRLAVAPGHKVEQKMSLTLFMKYGLVMNVTPRDLTPILAKIAKFGKIESCAGEHLINNGIHQPEAIAVNGIA*

>rf 1 sample sequence 4

MFWTGKLVLLEDMDASTGMMIVSIVAAYLLWFKSITKSMKGPKGPKMWPIVGSLPGLLENGTRMHEWIAENLRACAGTYQTCIFAIPFLARKQGLVTVTCDPKNLEHILKVRFDNYPKGPTWQAVFHDLLGEGIFNSDGDTWLFQRKTAALEFTTRTLRQAMGRWVNRAIKNRFCPILEMAQVQGKPVDLQDLLLRLTFDNICGLAFGKDPETLSPELPENNFATSFDRATEASLHRFIMPEFVWKLKKMLGLGMEVSLSHSLKQVDDYMTDVINTRKLELLNHQDGGPKHDDLLSRFMKKKESYSNKFLQHVALNFILAGRDTSSVALSWFFWLVSLNPRVEEKILIELCTVLAETRGNDTSKWLEEPLVFEEVDRLTYLKAALSETLRLYPSVPEDSKHVICDDYLPDGTFVPAGSNITYSIYSTGRMKFIWGEDCLEFKPERWMSQDGNKYQVQDAFRFVAFNAGPRICLGKDLAYLQMKSIAAAVLLRHRLAVAPGHKVEQKMSLTLFMKYGLVMNVTPRDLTPILAKFGKIESCAGEHLINNGIHQPEAITVNGIA*

>rf 1 sample sequence 4

MEKEVFFKILGFSFLVSIIMIRVVAYLWLRPRKIEEHFAKQGIRGPPYKFFIGNAKEIVSLMLKASSQTMPYSSHNILPRVLSFYHHWKKIYGATFLVWFGPTPRLAVADPDLIREIFTTKSEFYEKNEAHPLIRQLEGDGLLSLKGEKWAHHRKIITPTFHMENLKNGGIEIEVSEWFQTLTEDIVAQTAFGRSYEQGKAIFRFLPTRRNIKSWKLDTEIKKSLMKLIQERTEDWGKEMQENGPKDLLGLMIQASMKESSLSSSINSPLNHNSAINSSMITANDIAEECKTFFFAGEQTTSNLLTWTTVLLAMHPQWQDLARDEVIKVCGSRDIPSKDDLAKLKMADVDLGGCKIPLGTEVLIPILAVHHDQAIWGNDANEFNPARFSEGVARAAKHPVAYIPFGLGVRQCIGQNLAILQTKLTLAIILQRFTLRLSPQYKHAPTVLMLLHPQYGAPIIFQQRLSNPTIVKSS*

>rf 1 sample sequence 5

MDIAIALLLFTAITCYLLWFTFISRSLKGPRVWPLLGSLPGLIENSERMHDWIVDNLRACGGTYQTCICAVPFLARKQGLVTVTCDPKNLEHILKTRFENYPKGPTWQAVFHELLGQGIFNSDGDTWLFQRKTAALEFTTRTLRQAMARWVNRAIQLRFCPILKTAQLENKPVDLQDLLLRLTFDNICGLAFGKDPQTLAPGLPENTFASAFDRATEASLQRFILPEVIWKLKKWLGLGMEVSLNRSLVQLDKYMSDIINTRKLELMSQQKDGNPHDDLLSRFMKKKESYTDKFLQHVALNFILAGRDTSSVALSWFFWLVIQNPVVEQKILHEICTVLIETRGSDTSSWLDEPLAFEEVDRLTYLKAALSETLRLYPSVPEDSKHVVVDDVLPDGTFVPAGSSITYSIYSAGRMKTTWGEDCLEFKPERWLTPDGKKFVMHEQYKFVAFNAGPRICLGKDLAYLQMKSVAAAVLLRHRLTVAPGHKVEQKMSLTLFMKDGLKVNLRPRNLTPLVTSIKKEREVQLVQKLHGDKDECS*


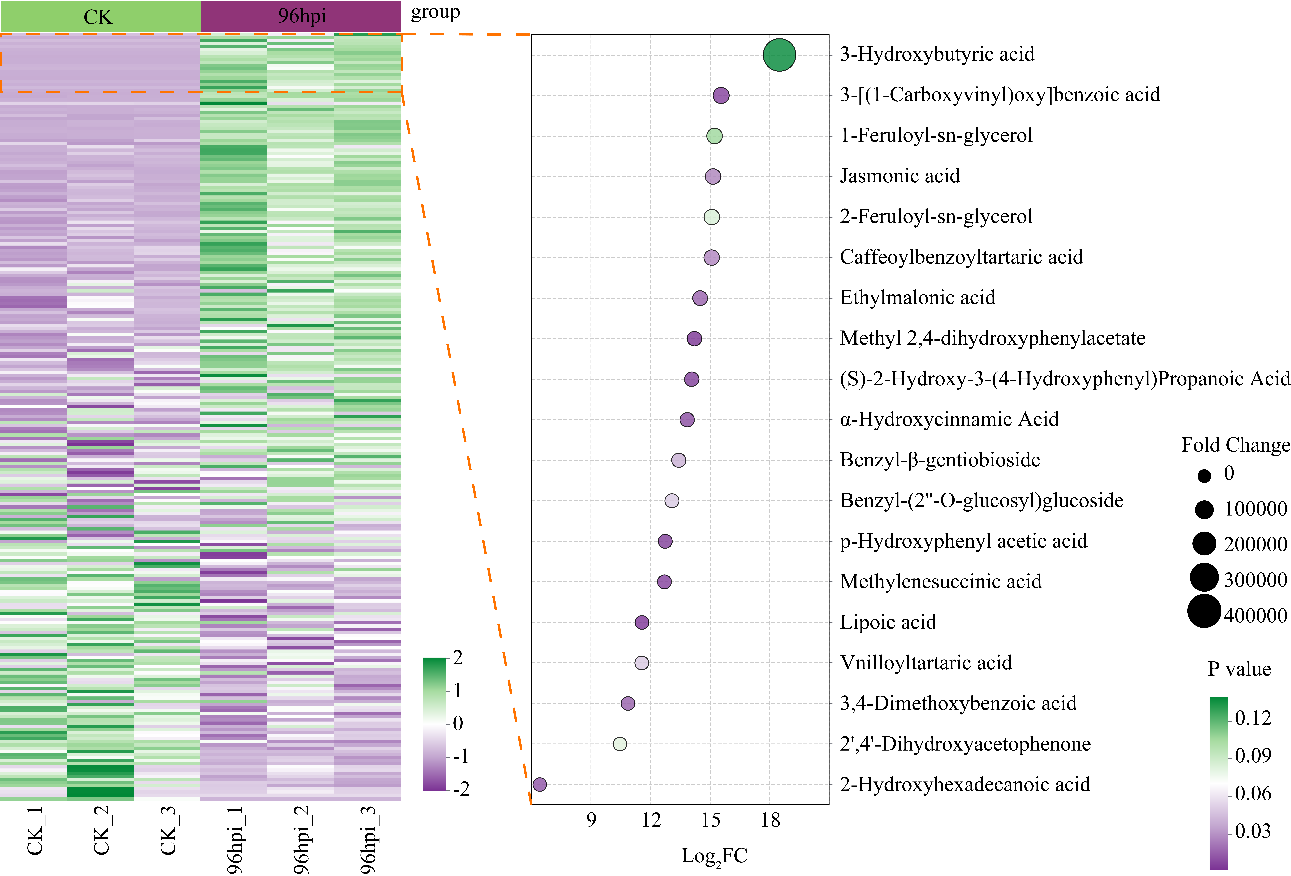


**Figure S1 Metabolomic differences between diseased and non-diseased tissue.**


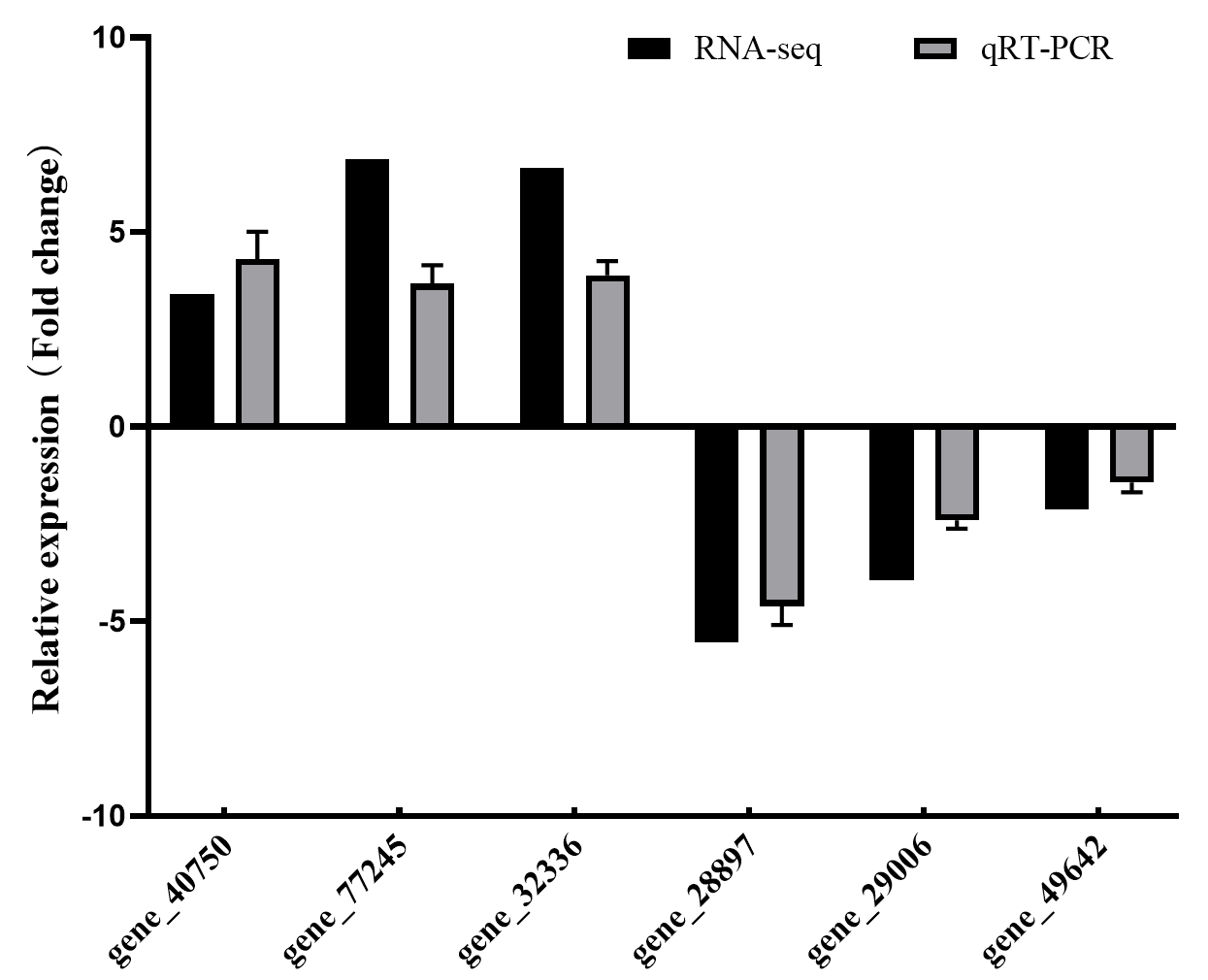


**Figure S2. A comparative analysis between RNA-seq and qPCR data confirmed the expression patterns of six selected genes at 4 h. (three up regulated genes and three down-regulated genes).**


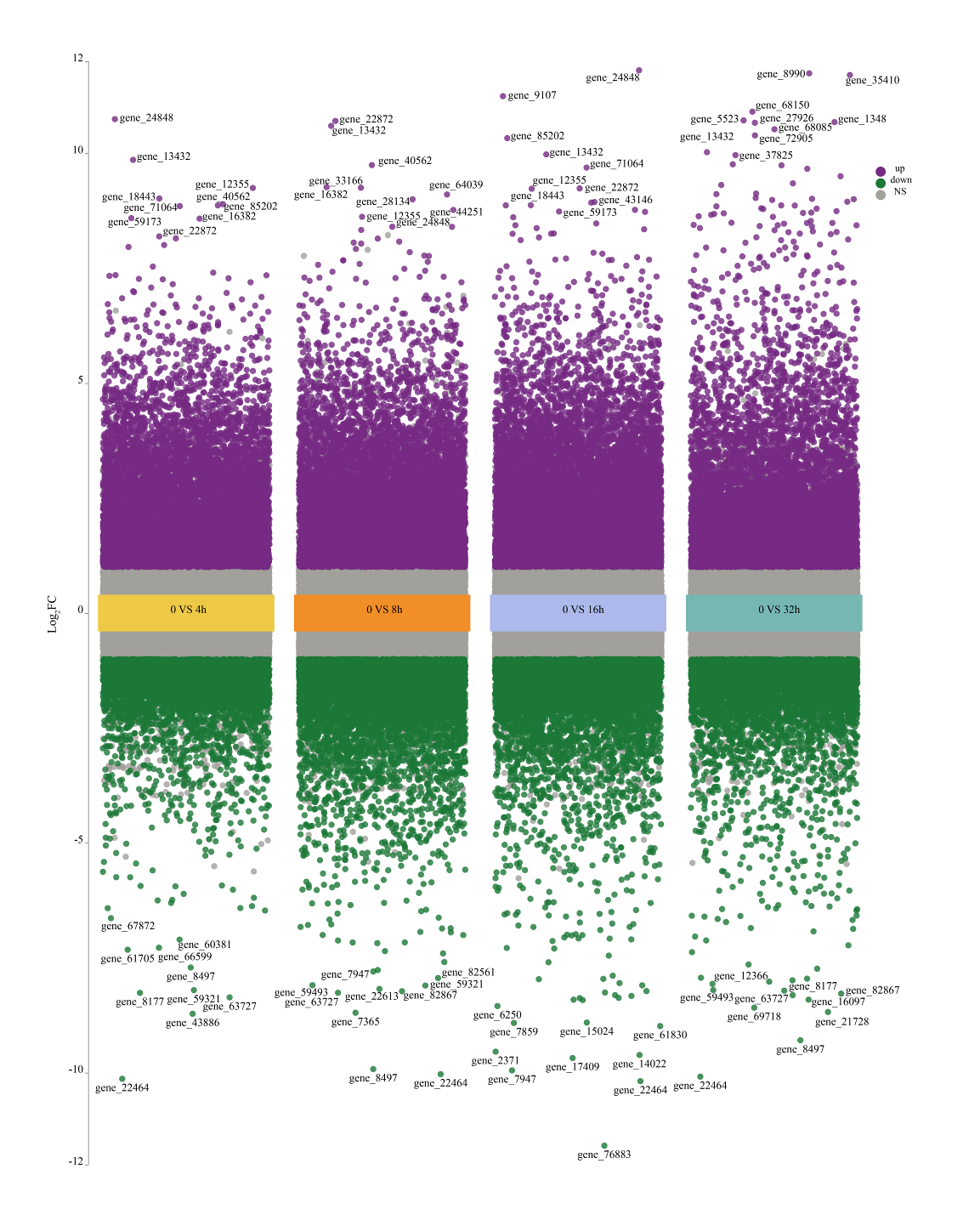


**Figure S3. Differential expression analysis of genes after veratric acid treatment.**

The y-axis represents the log₂-transformed fold change in gene expression between groups, reflecting the magnitude of differential expression. Gene IDs of the top 10 DEGs (differentially expressed genes) in each comparison group are labeled on the plot. Genes are color-coded to indicate expression status: upregulated (purple), downregulated (green), or unchanged (gray) between groups. The color gradient highlights statistical significance and directionality of expression changes, with purple and green dots denoting genes with significant fold changes and gray dots representing non-significant differences. This visualization identifies key genes responsive to veratric acid treatment, providing insights into temporal expression dynamics and potential functional candidates for further analysis.


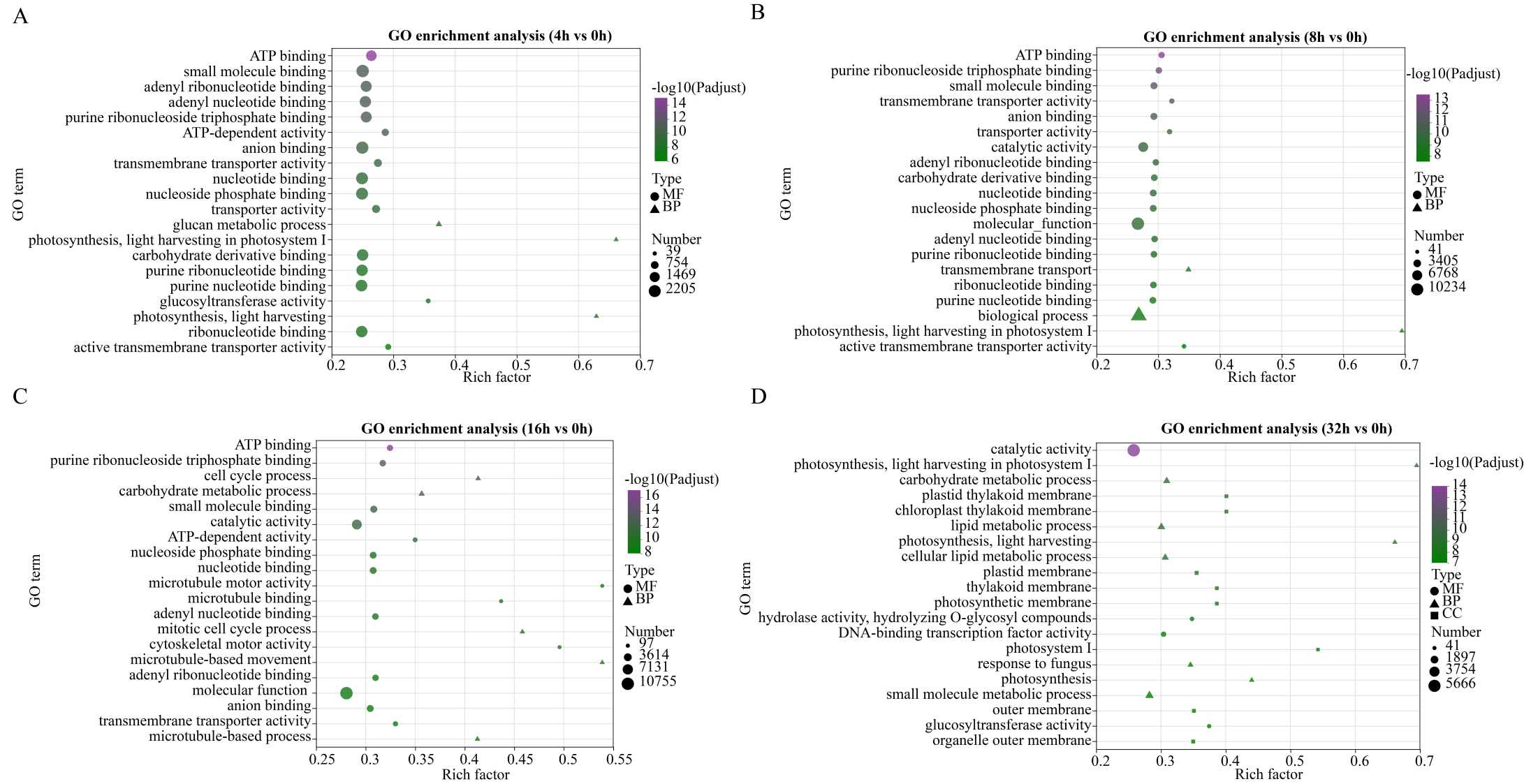


**Figure S4. GO Enrichment Analysis of Differentially Expressed Genes (DEGs) after Veratric Acid Treatment..**

(A–D) Bubble plots of enriched GO pathways following treatment with veratric acid for 4 h (A), 8 h (B), 16 h (C), and 32 h (D). The x-axis represents the Rich factor, defined as the ratio of the number of genes/transcripts enriched in a specific GO term (sample number) to the total number of annotated genes/transcripts in the background (background number), with a higher Rich factor indicating greater enrichment significance. The size of each point corresponds to the number of genes/transcripts in the GO term, while the color gradient reflects the range of Padjust values (adjusted p-values for multiple testing). Different shapes denote distinct GO types: circles for Molecular Function (MF), triangles for Biological Process (BP), and squares for Cellular Component (CC). Only the top 20 enriched terms with Padjust < 0.05 are displayed to highlight the most significant functional categories associated with veratric acid treatment at each time point


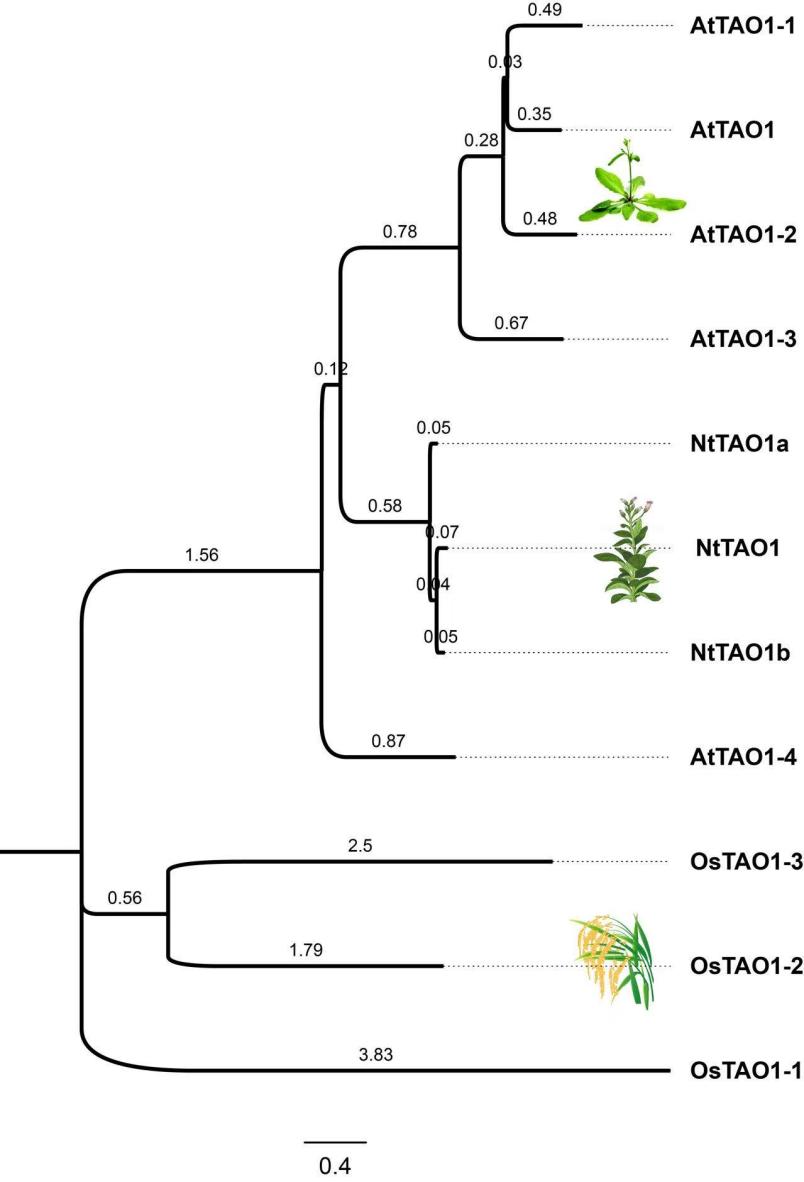


**Figure S5 Phylogenetic Tree of NtTAO1 Protein and Its Homologs in Other Species Constructed by Neighbor-Joining Method**

The genomic sequence of NtTAO1 (gene_28897) was retrieved from the tobacco K326 genome database (https://solgenomics.net/tools/blast/?db_id=235). The nucleotide sequence was analyzed using the ORF finder tool (https://www.ncbi.nlm.nih.gov/orffinder/) to identify the open reading frame (ORF) and translate the deduced amino acid sequence. The amino acid sequence of NtTAO1 was then subjected to a BLASTp search (https://blast.ncbi.nlm.nih.gov/) to identify homologous TAO1 proteins from other plant species, which were subsequently downloaded for further analysis.

A phylogenetic tree was constructed using the Neighbor-Joining (NJ) method in MEGA X software. The analysis parameters were set as follows: the bootstrap method with 1000 replicates was selected to assess the reliability of the phylogenetic tree topology. The evolutionary distances were computed using the Poisson correction method, and all positions containing gaps and missing data were eliminated from the dataset (complete deletion option).


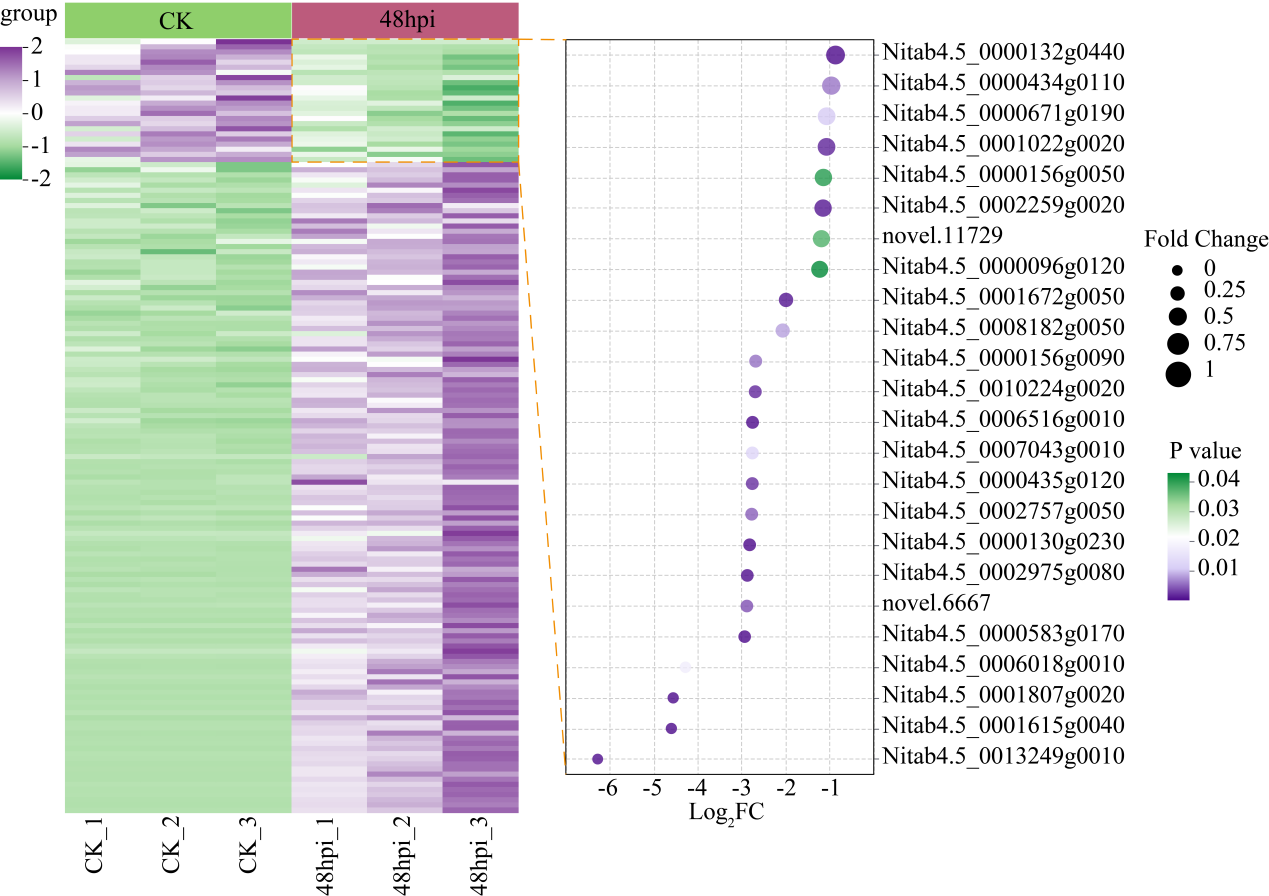


**Figure S6 Heatmap of Genes Related to P450 Protein Expression**

**
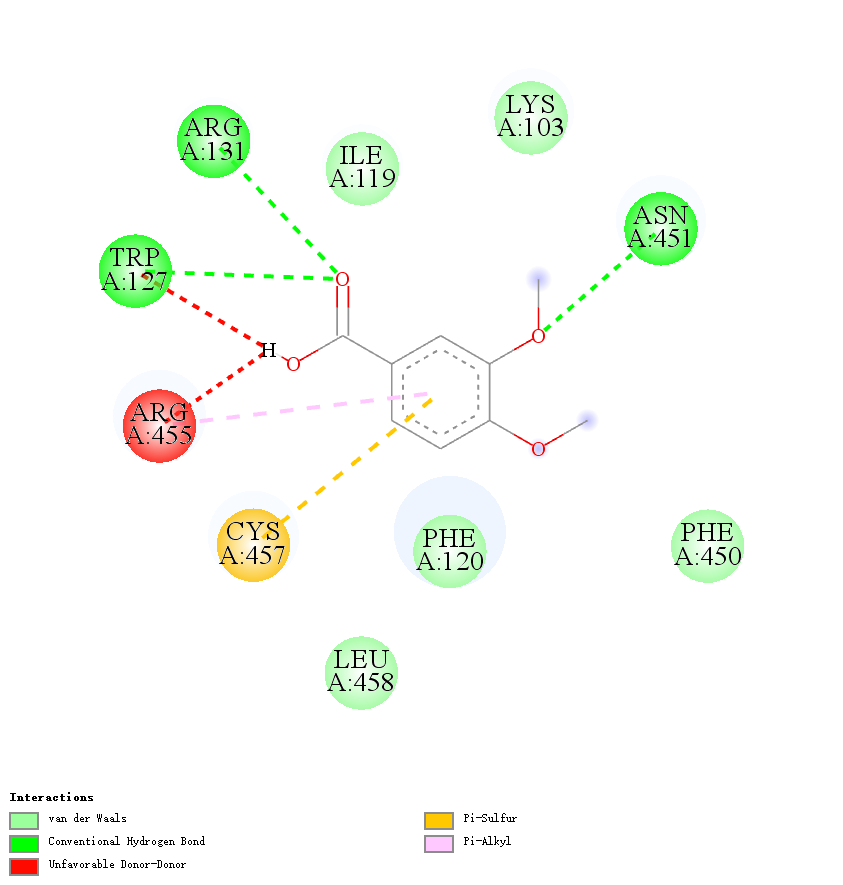
**

**Figure S7 Molecular Docking Diagram of P450 Protein 1 with VA**


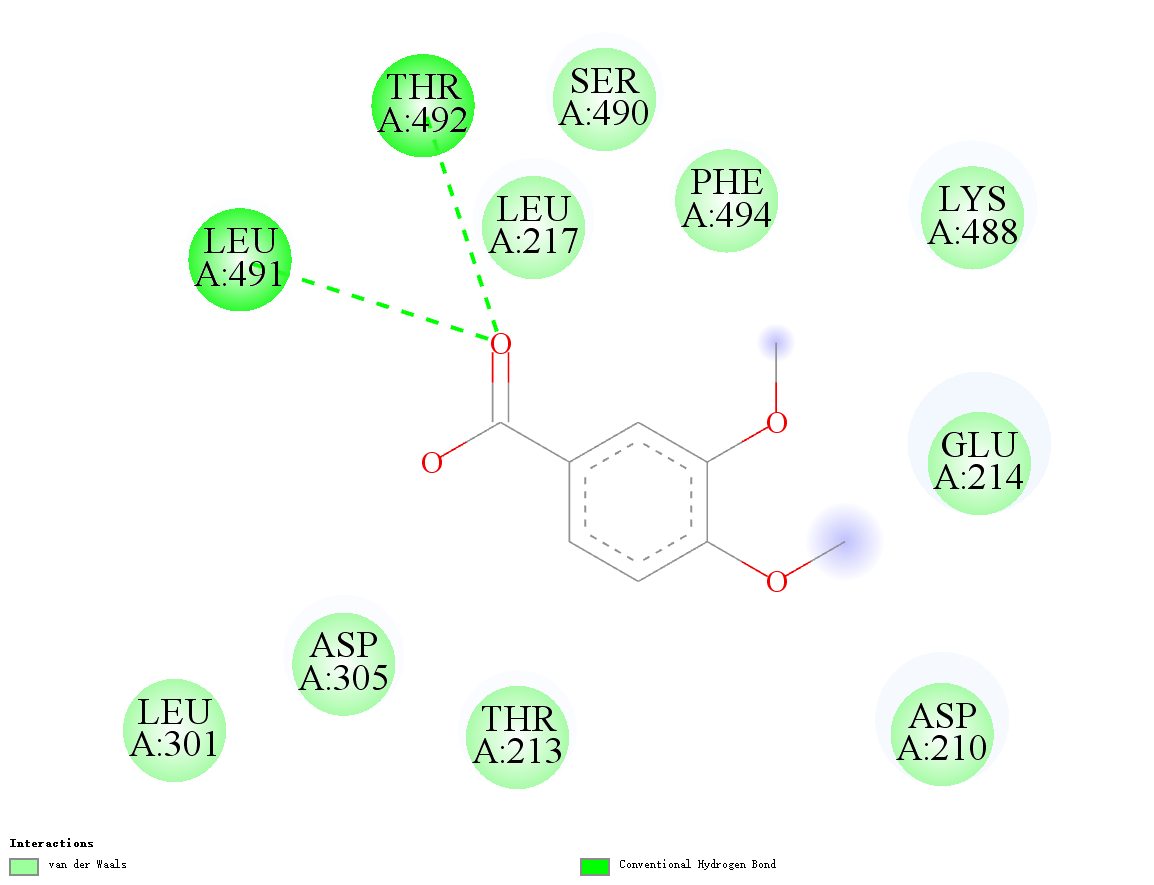


**Figure S8 Molecular Docking Diagram of P450 Protein 2 with VA**


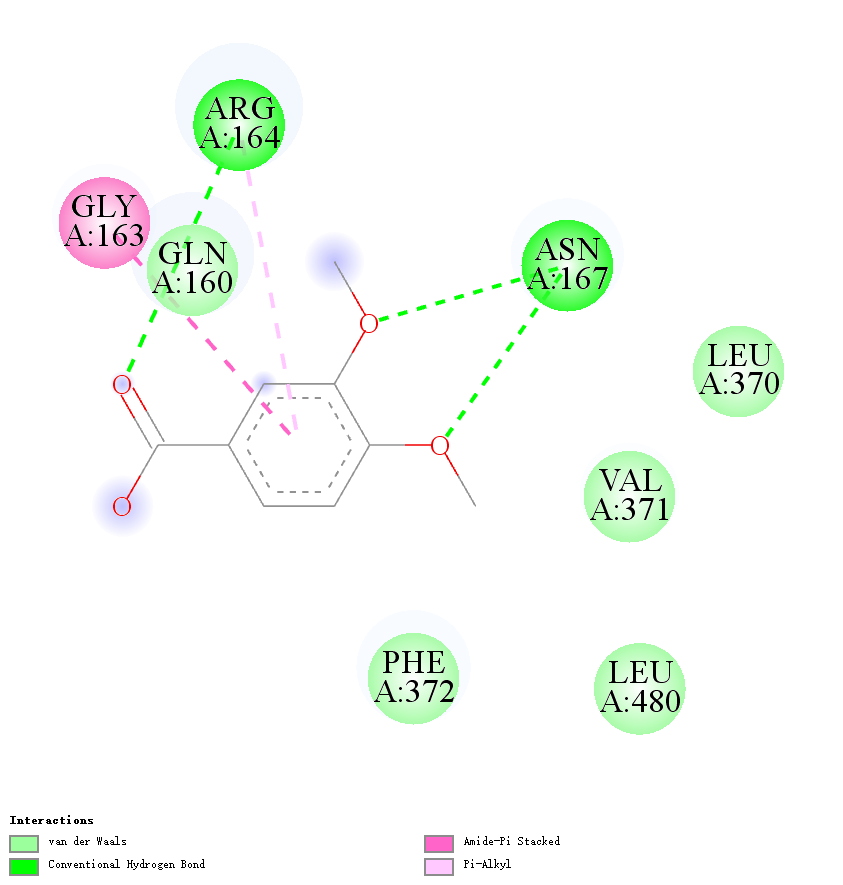


**Figure S9 Molecular Docking Diagram of P450 Protein 3 with VA**


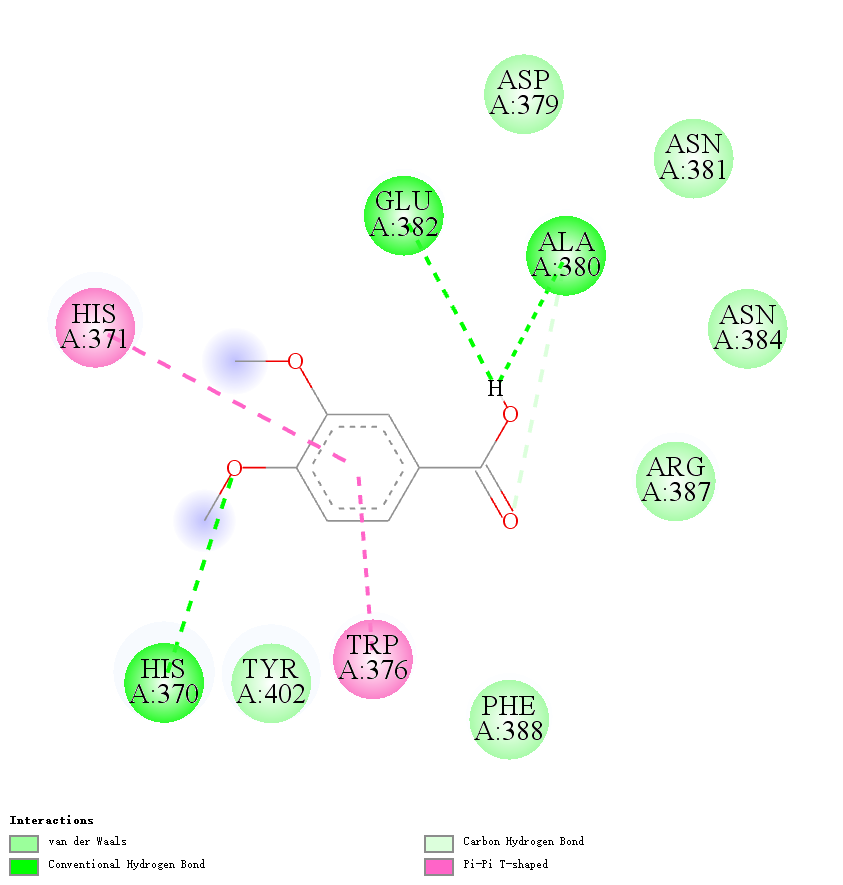


**Figure S10 Molecular Docking Diagram of P450 Protein 4 with VA**


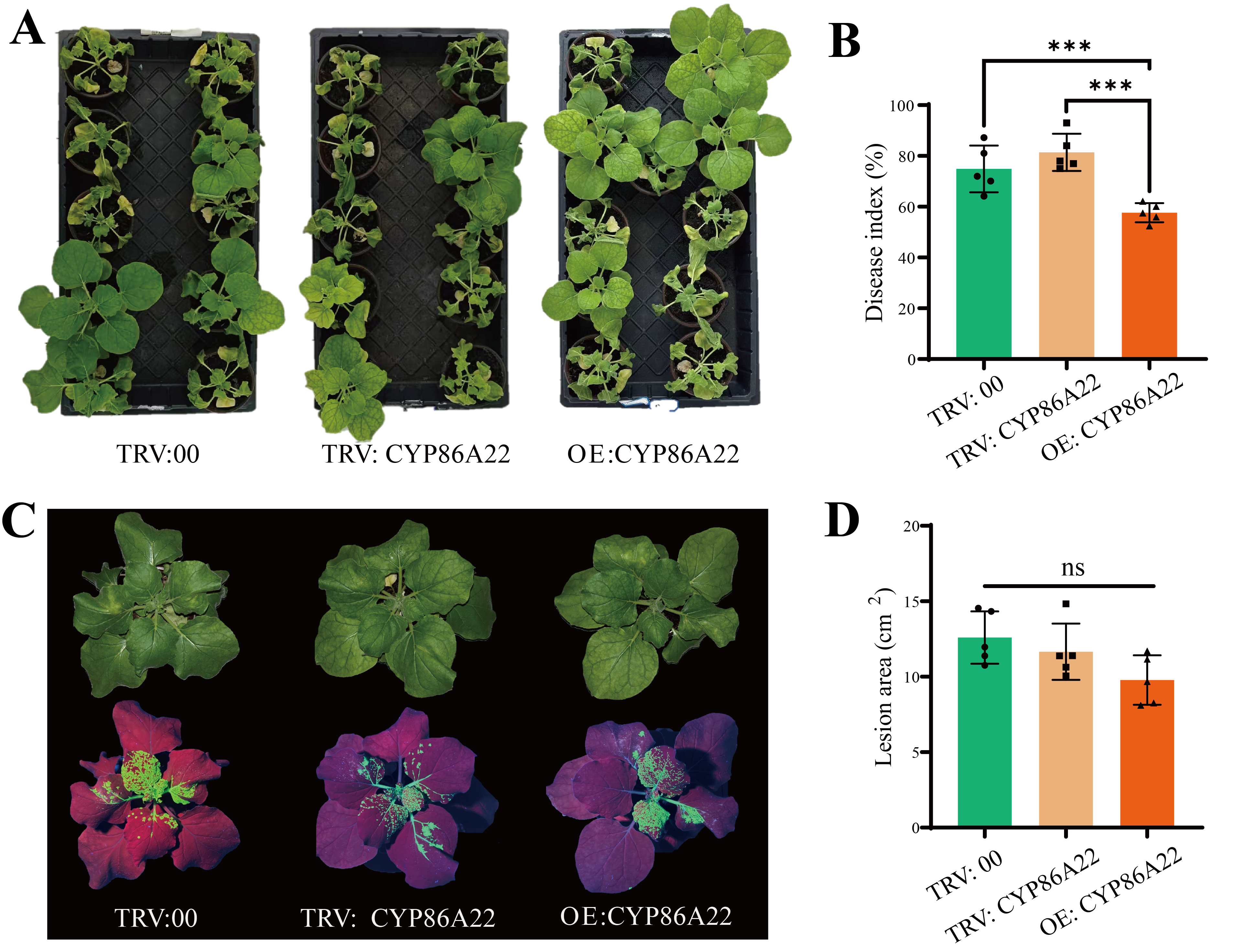


**Fig S11. Pathogenicity assay of CYP86A22-silenced and Overexpression plants.**

**A&B, Susceptibility to bacterial wilt; C&D, Susceptibility to TMV.**

**It can be observed that silencing of CYP86A22 did not significantly alter disease resistance or susceptibility.**

**
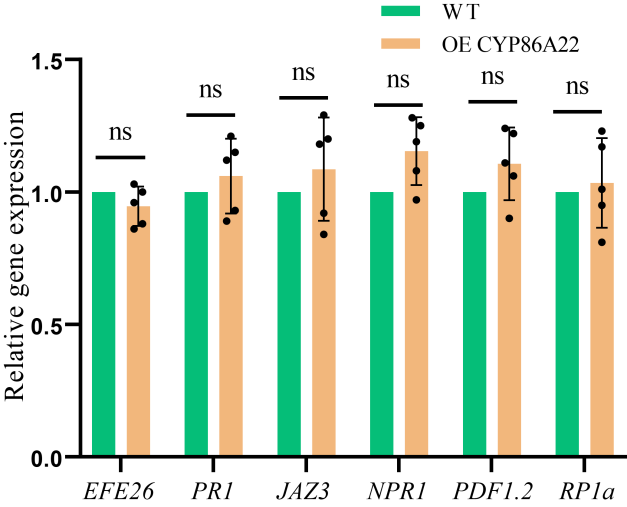
**

**Fig S12. Expression levels of disease resistance-related genes in *N. benthamiana* overexpressing *CYP86A22.***

**
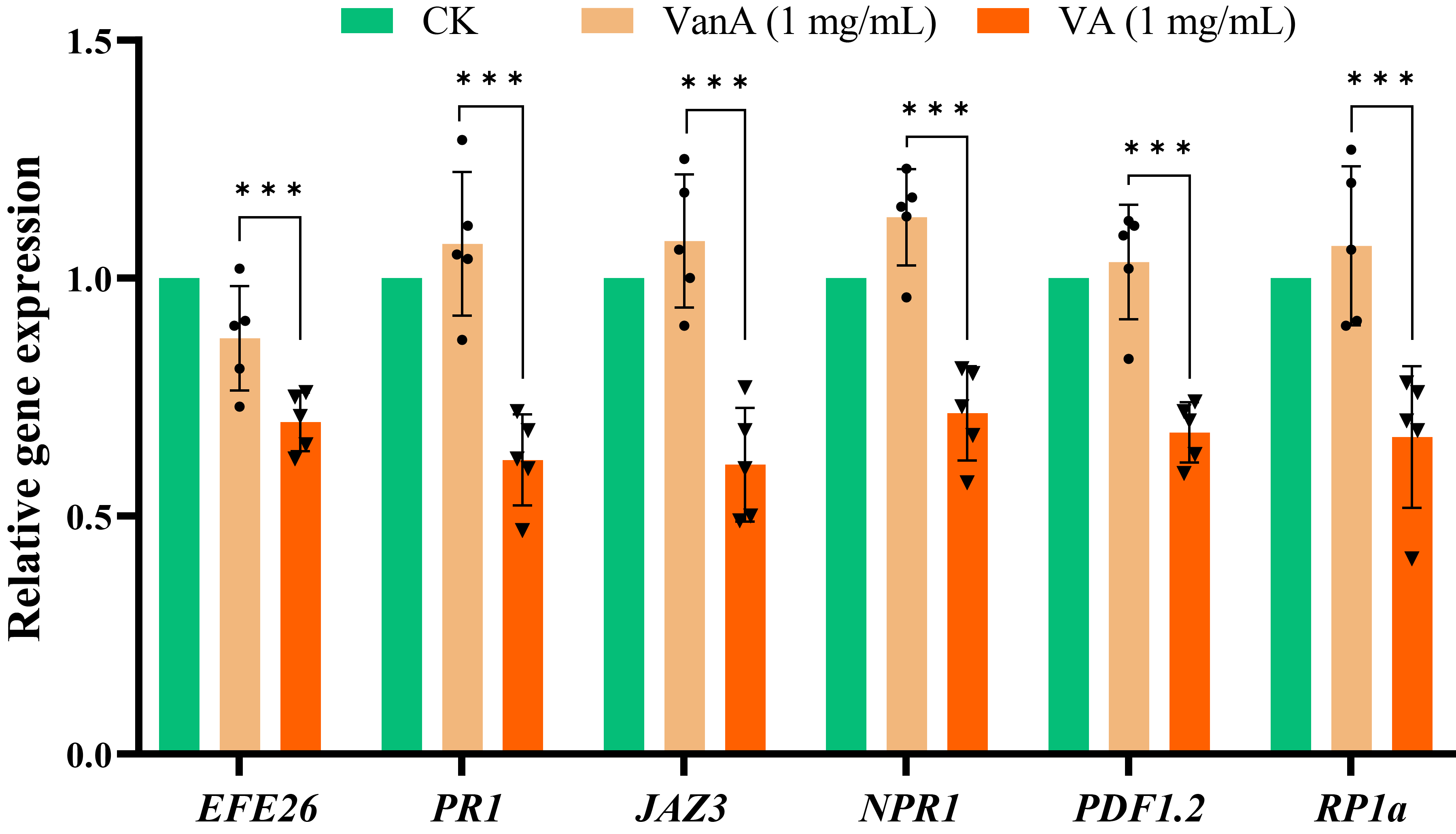
**

Fig S13. Expression levels of disease resistance-related genes in *N. benthamiana* under VA and VanA treatments.

**
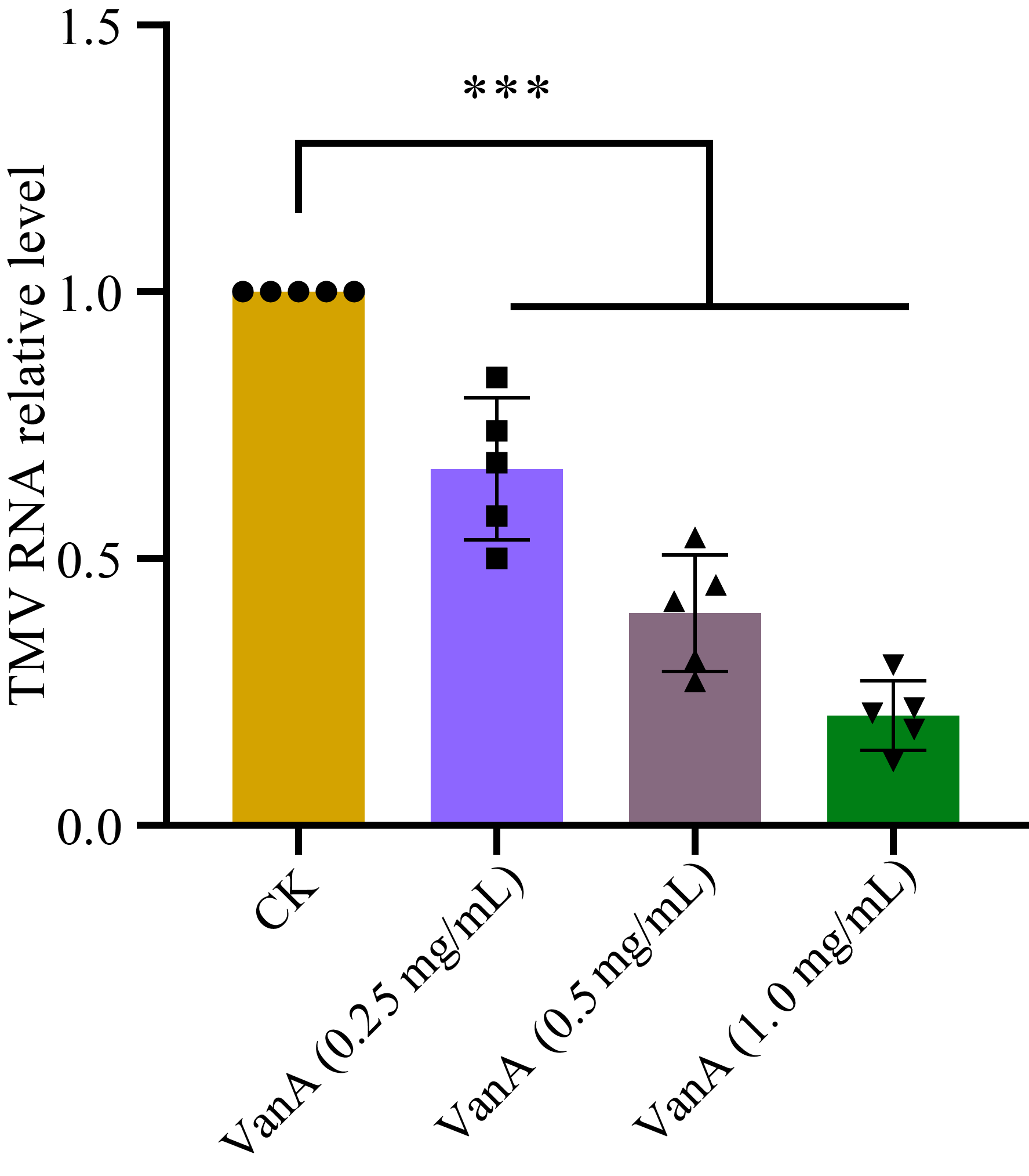
**

Fig S14. TMV accumulation after treatment with different concentrations of VanA (3 dpi).


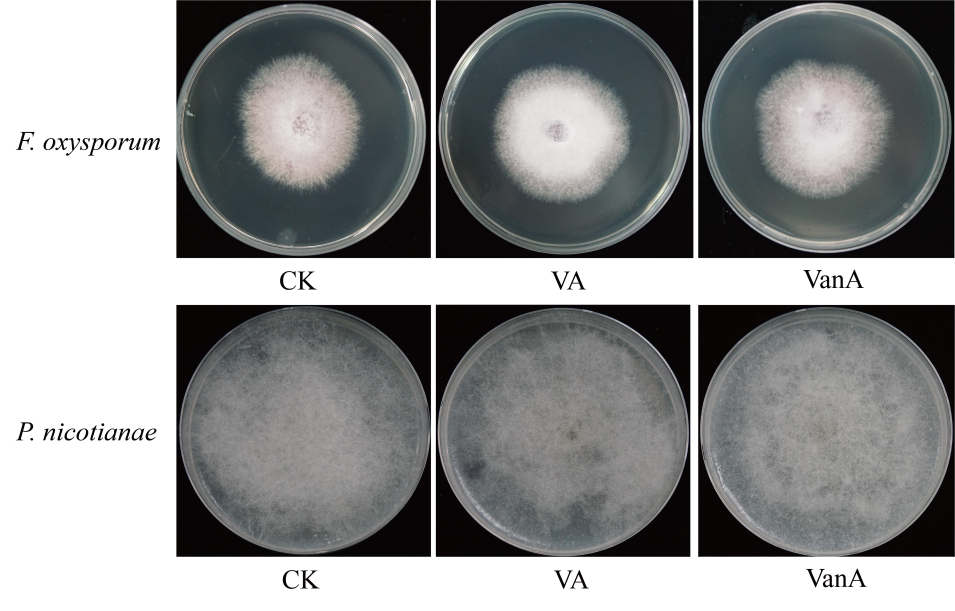


Fig S15. No significant inhibitory effects against fungi and oomycetes were observed at a concentration of 1 mg/mL.


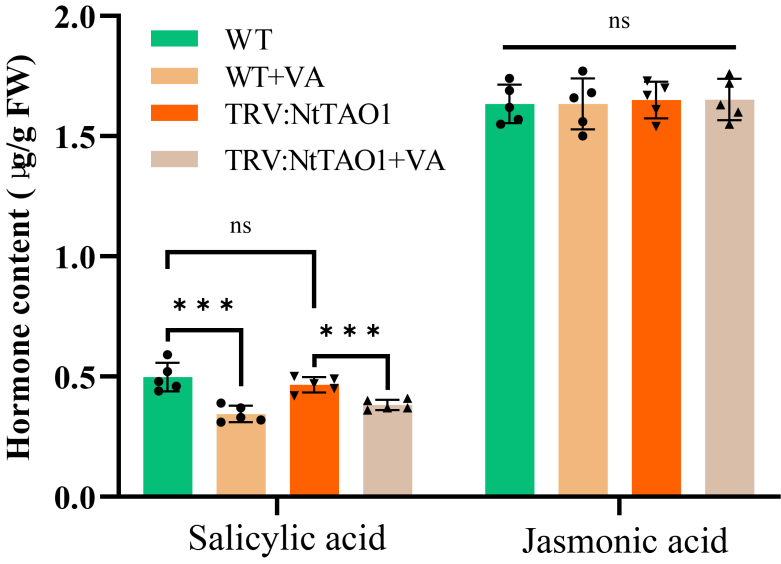


Fig S16. Changes in salicylic acid (SA) and jasmonic acid (JA) contents in wild-type and NtTAO1-silenced *N. benthamiana* after VA treatment.


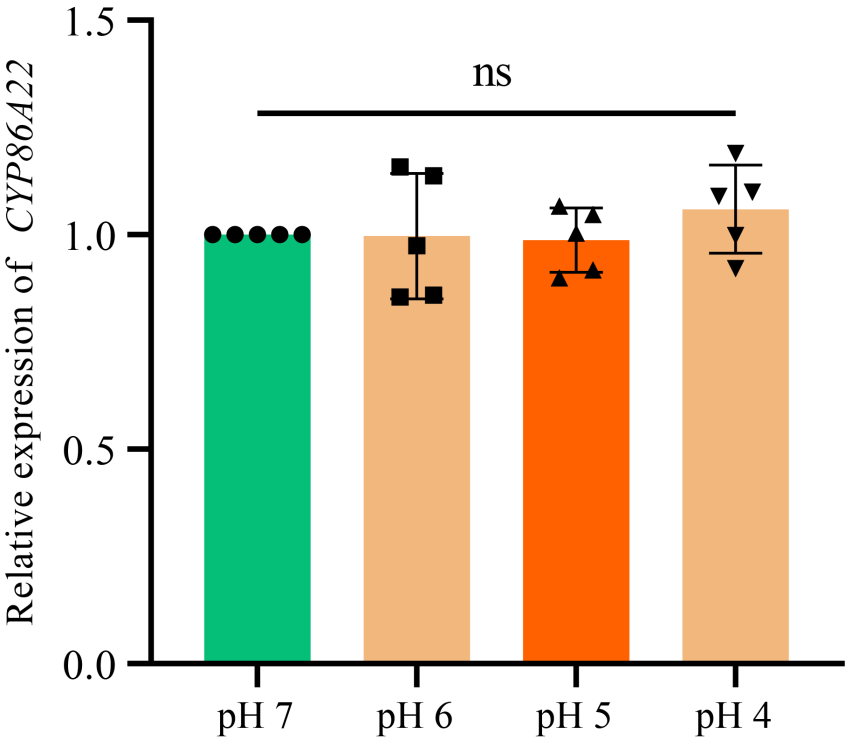


Fig S17 Acidified soil does not induce the expression of *CYP86A22*


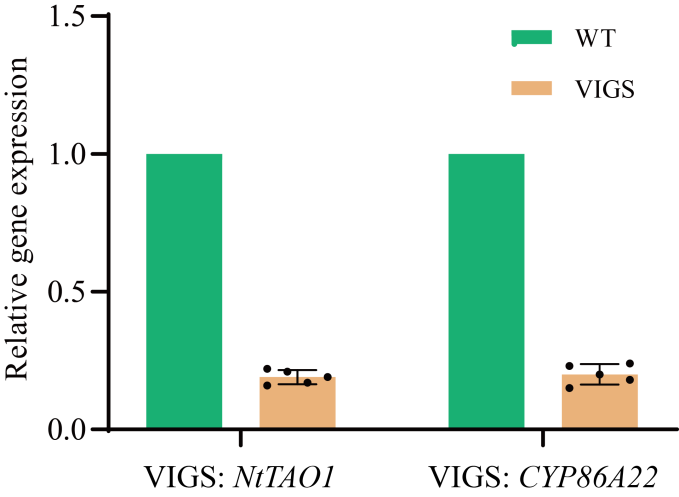


Fig. S18 VIGS-Mediated Gene Silencing Efficiency


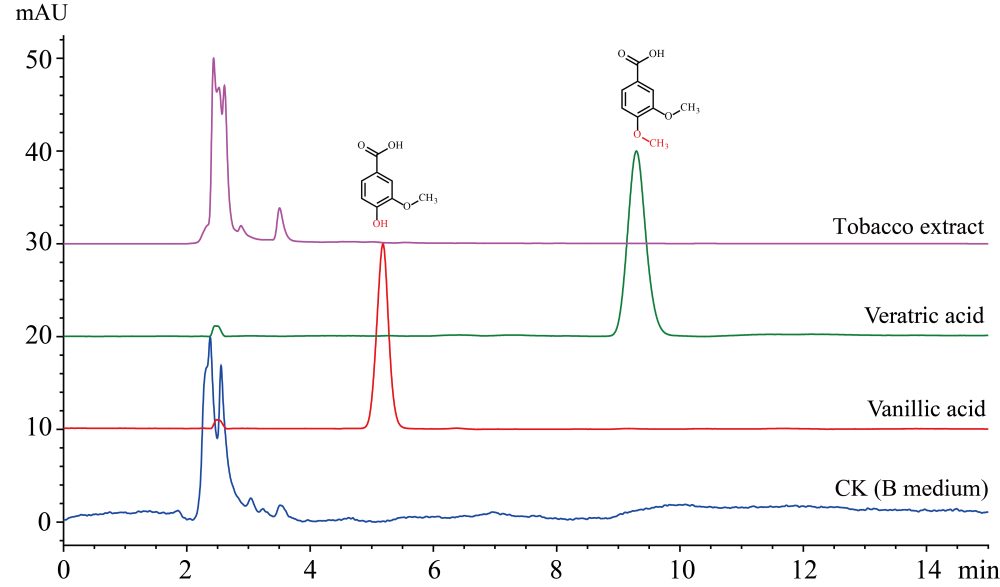


Fig. S19 HPLC analysis of VA and VanA contents in culture supernatants of *R. solanacearum*.

Table S1 EC_50_ of tested compounds against *R. solanacearum*

| Compound | EC_50_(mg/mL) | Toxicity regression eq. (Y = a + bX) | R^2^ |
| --- | --- | --- | --- |
| Veratrol acid | 0.746 | y=2.7 + 0.34X | R^2^=0.955 |
| 3-Hydroxybutyric acid | 0.65 | y=2.09 + 3.05X | R^2^=0.919 |
| Ethylmalonic acid | 0.664 | y=2.11 + 3.04X | R^2^=0.923 |
| p-Hydroxyphenylacetic acid | 1.052 | y=2.21 + 2.16X | R^2^=0.928 |
| Hydroxybenzoic acid | 0.695 | y=2.44 + 3.3X | R^2^=0.918 |
| Itaconic acid | 0.584 | y=1.78 + 2.94X | R^2^=0.954 |
| Vanillic Acid | 0.678 | y=1 + 4.66X | R^2^=0.964 |

Table S2: The primer sequences used in this study.

| Primer name | Sequence 5'-3' |
| --- | --- |
| RT-40750F | TGTCAAACCAGAACGCTAGC |
| RT-40750R | ATAAGCGAAGGCCGTTTTGG |
| RT-77245F | TTACAACGGCGTCTTCACAC |
| RT-77245R | AAAGCACATGCAAGCGTCTC |
| RT-32336F | ATCGCGTCGAAGATGTTGTC |
| RT-32336R | TGGCACACAAACTCGTGATC |
| RT-28897F | TGCAAGCAACTCAGAAGCAC |
| RT-28897R | TCTCAGTCAGACTTGAGCAACC |
| RT-29006F | TGCTGAAAGTGAGTCCTTGC |
| RT-29006R | GCTTCAGTTGATGTTGCTGAAAG |
| RT-49642F | ACAGTTGGCGAAGGAACAAC |
| RT-49642R | CAAGATGCAAAACGCGCAAC |

Table S3 Binding Energy Obtained from Molecular Docking

| Gene Identifier | Binding Energy(kcal/mol） |
| --- | --- |
| Nitab4.5_0013249g0010（1#） | -6.1 |
| Nitab4.5_0000583g0170（2#） | -4.9 |
| novel.6667（3#） | -5.2 |
| Nitab4.5_0002975g0080（4#） | -6.4 |
| Nitab4.5_0008182g0050 | -5.6 |
| Nitab4.5_0001615g0040 | -2.1 |
| Nitab4.5_0000130g0230 | -2.7 |
| Nitab4.5_0000096g0120 | -3 |
| Nitab4.5_0001807g0020 | -4.4 |
| Nitab4.5_0000156g0050 | -4.2 |
| Nitab4.5_0002259g0020 | -2.4 |
| Nitab4.5_0002757g0050 | -3.7 |
| Nitab4.5_0000671g0190 | -4.2 |
| Nitab4.5_0007043g0010 | -3.1 |
| Nitab4.5_0001022g0020 | -4.8 |
| Nitab4.5_0000435g0120 | -2 |
| Nitab4.5_0006516g0010 | -3.5 |
| Nitab4.5_0000434g0110 | -3.8 |
| Nitab4.5_0001672g0050 | -4.3 |
| Nitab4.5_0006018g0010 | -2.5 |
| Nitab4.5_0010224g0020 | -4.1 |
| Nitab4.5_0000132g0440 | -3.4 |
| novel.11729 | -4.5 |
